# The ubiquitin ligase ZNRF1 promotes caveolin-1 ubiquitination and degradation to modulate inflammation

**DOI:** 10.1038/ncomms15502

**Published:** 2017-06-08

**Authors:** Chih-Yuan Lee, Ting-Yu Lai, Meng-Kun Tsai, Yung-Chi Chang, Yu-Hsin Ho, I-Shing Yu, Tzu-Wen Yeh, Chih-Chang Chou, You-Sheng Lin, Toby Lawrence, Li-Chung Hsu

**Affiliations:** 1 Institute of Molecular Medicine, National Taiwan University, No. 7 Chung San South Road, Taipei 10002, Taiwan; 2 Department of Surgery, National Taiwan University Hospital, No. 7 Chung San South Road, Taipei 10002, Taiwan; 3 Laboratory Animal Center, College of Medicine, National Taiwan University, No. 7 Chung San South Road, Taipei 10002, Taiwan; 4 Institut National de la Santé et de la Recherche Médicale (INSERM), U1104, 13288 Marseille, France

## Abstract

Caveolin-1 (CAV1), the major constituent of caveolae, plays a pivotal role in various cellular biological functions, including cancer and inflammation. The ubiquitin/proteasomal pathway is known to contribute to the regulation of CAV1 expression, but the ubiquitin ligase responsible for CAV1 protein stability remains unidentified. Here we reveal that E3 ubiquitin ligase ZNRF1 modulates CAV1 protein stability to regulate Toll-like receptor (TLR) 4-triggered immune responses. We demonstrate that ZNRF1 physically interacts with CAV1 in response to lipopolysaccharide and mediates ubiquitination and degradation of CAV1. The ZNRF1–CAV1 axis regulates Akt–GSK3β activity upon TLR4 activation, resulting in enhanced production of pro-inflammatory cytokines and inhibition of anti-inflammatory cytokine IL-10. Mice with deletion of ZNRF1 in their hematopoietic cells display increased resistance to endotoxic and polymicrobial septic shock due to attenuated inflammation. Our study defines ZNRF1 as a regulator of TLR4-induced inflammatory responses and reveals another mechanism for the regulation of TLR4 signalling through CAV1.

## Abstract (web summary)

Caveolae and their major constituent Caveolin-1 (CAV1) play an important role in signalling pathways involved in inflammation, but regulators of CAV1 protein stability are unknown. Here, the authors show that E3 ubiquitin ligase ZNRF1 induces degradation of CAV1 in response to TLR4 activation, and mediates the pro-inflammatory response both *in vitro* and *in vivo*.

Inflammation is an important component of innate immunity and the host response to pathogen infection. Upon infection, innate immune cells, including macrophages and dendritic cells, use Pattern-Recognition Receptors (PRRs), mainly Toll-like receptors (TLRs), to detect conserved microbial molecules termed pathogen-associated molecular patterns (PAMPs) or danger-associated molecular patterns (DAMPs), which are released from injured or dead cells. The innate immune cells then mount a defence response to clear microbes or damaged cells and coordinate the adaptive immune response^1^. Excess activation of pro-inflammatory responses has been associated with many inflammatory diseases, including atherosclerosis, rheumatoid arthritis and sepsis^2^. Thus, the proper regulation of the inflammatory response is an important issue for the treatment of inflammatory diseases.

TLRs are crucial components of innate immunity. By recognizing conserved pathogen components, TLRs activate specific signalling pathways and inflammatory responses. The localization of TLRs dictates which adaptor proteins it interacts with and subsequent downstream signalling and gene induction. Upon the binding of bacterial lipopolysaccharide (LPS), TLR4 and MD2 are recruited to phosphatidylinositol 4,5-bisphosphate [PtdIns(4,5)P_2_]-rich microdomains at the plasma membrane, where TLR4 associates with Myeloid differentiation primary response gene-88 (MyD88) via the sorting adaptor MyD88-adapter-like (Mal), leading to early IKK-NF-κB and Mitogen-activated protein kinase (MAPK) activation and the production of pro-inflammatory cytokines^3^,^4^. TLR4 and CD14 are then engulfed into the endosomal compartment to trigger secondary signalling by recruiting Toll/IL-1 receptor domain-containing adaptor-inducing IFNβ (TRIF) via the sorting adaptor TRIF*-*related adaptor molecule (TRAM), resulting in interferon regulatory factor 3 (IRF3) activation and the induction of type I interferon (IFN)^5^. TLR4 endocytosis is mediated by the clathrin-dependent and lipid rafts/caveolae-dependent endocytic pathways, and both internalization routes are dependent on the GTPase dynamin^6^. However, the molecular mechanism underlying the sorting of activated TLR4 and its downstream signalling is not well understood.

Lipid rafts are higher-order membrane microdomains regarded as assemblies of glycosphingolipids and cholesterol. In addition, they are dynamic and resistant to non-ionic detergents^7^. Caveolae, a special subset of lipid rafts, are small, flask-shaped invaginations (60–80 μm in diameter) of the plasma membrane. The functions of caveolae include endocytosis, phagocytosis, sorting of vesicles and the regulation of various signalling pathways^8^. The major constituent of caveolae is the protein CAV1, which is essential for caveolae biogenesis and the regulation of caveolae-mediated cellular functions^9^. Interestingly, CAV1 is also a negative regulator of diverse cellular signalling pathways^10^. Given the importance of CAV1 in caveolae formation and various cellular functions, the regulation of CAV1 expression has been the subject of extensive study. However, most of these studies have focused on the transcriptional control of CVA1 expression, and its post-transcriptional regulation remains to be identified.

Accumulating evidence indicates that lipid rafts/caveolae play an important role in the signalling pathways involved in the activation of both innate and adaptive immune responses^11^. TLR2 and TLR4 were shown to translocate to lipid rafts, where they assemble with the adaptor proteins Mal-MyD88 and downstream signalling molecules upon ligand binding^10^,^12^. However, the overexpression of CAV1 was recently reported to exert anti-inflammatory effects in response to LPS, although the underlying mechanism has yet to be identified^13^.

In this study, we identify a ring-type E3 ubiquitin ligase, zinc and ring finger 1 (ZNRF1) (previously identified as a nerve injury-induced gene^14^), which promotes CAV1 degradation during inflammation through the ubiquitin/proteasome system. We discover that, in macrophages, ZNRF1 associates with and promotes CAV1 degradation upon TLR4 engagement. The depletion of ZNRF1 in macrophages leads to increased PI3K-Akt activity in response to LPS, which then blocks GSK3β activation, resulting in an attenuation of the expression of genes related to inflammation and the induction of anti-inflammatory cytokine IL-10, respectively. In addition, mice with a myeloid-specific-deletion of ZNRF1 are significantly resistant to both LPS- and cecal ligation and puncture (CLP)-induced sepsis and exhibit a reduced inflammatory response, demonstrating the positive regulatory role of ZNRF1 in TLR4-driven inflammatory response. Our results collectively reveal ZNRF1 as a novel regulator of the TLR4 signalling pathway through the control of CAV1 protein stability.

## Results

### ZNRF1 deficiency attenuates LPS-induced inflammation

Ubiquitination/deubiquitination is a crucial mechanism in the regulation of TLR-mediated immune responses. Several E3 ligases with roles in the control of TLR signalling pathways have been identified, such as TRAF3/6 and cIAP1/2 (ref. 15). We explored the GDS2047 microarray data set at the publicly accessible microarray database of the National Center for Biotechnology Information and identified an E3 ubiquitin ligase, ZNRF1, that was upregulated in macrophages upon LPS stimulation. To further explore the function of ZNRF1 in macrophages during inflammation, we knocked down the expression of the *Znrf1* gene in murine RAW264.7 macrophages using lentivirus-mediated shRNA transduction and stimulated these cells with a TLR4 agonist, LPS. ZNRF1 depletion significantly inhibited the mRNA expression of pro-inflammatory cytokines and chemokines, including TNF, IL-6, IL-1β, CCL5 and IFNβ, in response to LPS (Supplementary Fig. 1a). By contrast, the mRNA level of IL-10, a potent anti-inflammatory cytokine, was elevated in ZNRF1-silenced RAW264.7 macrophages compared to cells expressing control shRNA (shScr). Consistent with their mRNA expression, the levels of cytokines, including TNF, IL-6 and CCL5, were decreased in ZNRF1-deficent cells, whereas IL-10 was elevated after LPS stimulation (Supplementary Fig. 1b). Similar results were observed in human myelomonocytic THP1 cell-derived macrophages depleted of ZNRF1 (Supplementary Fig. 2a,b). These data suggest that ZNRF1 is an important regulator of TLR4-induced pro-inflammatory cytokine production.

**Figure 1:**
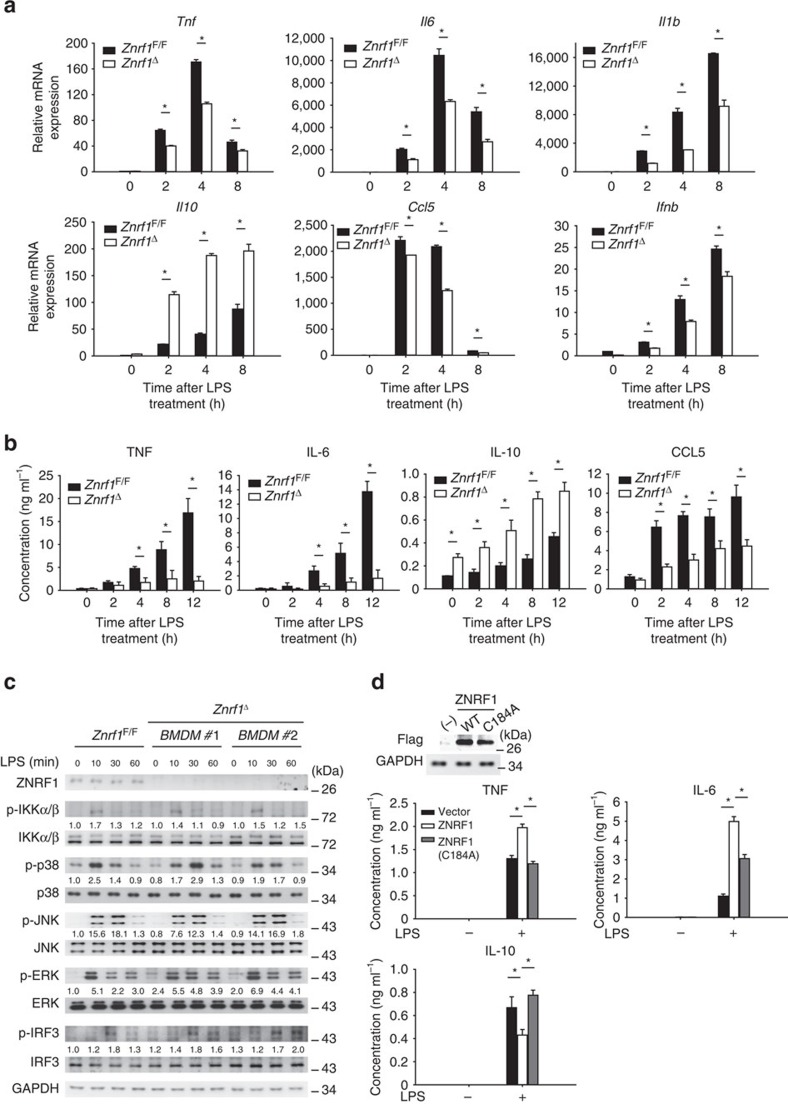
ZNRF1 deletion impairs LPS-induced production of inflammatory mediators in BMDMs. (**a**–**c**) BMDMs were harvested from wild-type (*Znrf1*^F/F^) or *Znrf1*^Δ^ mice and treated with LPS (100 ng ml^−1^) for the indicated times. (**a**) The expression of the indicated mRNAs was analysed by RT–qPCR. (**b**) The production of cytokines in supernatants was determined by ELISA. (**c**) The phosphorylation of MAPKs, IKKα/β, IRF3 as well as the indicated proteins in cell lysates was analysed by immunoblot analysis. The intensities of the bands are expressed as fold increases compared to those of untreated control cells after normalization to their unphosphorylated forms. (**d**) BMDMs from *Znrf1*^Δ^ mice were reconstituted with either Flag-tagged wild-type ZNRF1 or ZNRF1(C184A) mutant and stimulated with LPS (100 ng ml^−1^) for 8 h. The levels of the indicated cytokines in culture supernatants were measured by ELISA. *P<0.05 (Student's *t*-test). The data are representative of three independent experiments performed in triplicate (error bars, s.d.).

To test the significance of ZNRF1 in TLR4-mediated immune response *in vivo* we generated *Znrf1*^F/F^ (*loxP*-flanked *Znrf1*) mice by inserting two *loxP* sites into the first and third introns of the *Znrf1* gene through two sequential rounds of homologous recombination (Supplementary Fig. 3a). We crossed these mice with transgenic mice in which Cre recombinase is driven by an IFN-inducible Mx1 promoter. We deleted the *Znrf1* gene in cells of the myeloid lineage by administering polyinosinic-polycytidylic acid [poly(I:C)] to mice (called ‘*Znrf1*^Δ^' here). As a control, *Znrf1*^F/F^ mice received the same poly(I:C) administration schedule. The peripheral blood cell counts in *Znrf1*^F/F^ and *Znrf1*^Δ^ were similar (Supplementary Table 2). Bone marrow cells isolated from either *Znrf1*^Δ^ or *Znrf1*^F/F^ mice were differentiated into macrophages (BMDMs), and the BMDM numbers were comparable (Supplementary Fig. 3e). Consistent with the results in ZNRF1-silenced cells, the mRNA expression levels of TNF, IL-6, IL-1β, CCL5 and IFNβ were decreased, whereas *Il10* mRNA was elevated, in LPS-stimulated BMDMs from *Znrf1*^Δ^ mice compared to those from *Znrf1*^F/F^ mice (Fig. 1a). Accordingly, the levels of cytokines, including TNF, IL-6 and CCL5, were decreased in *Znrf1*^Δ^ BMDMs, whereas IL-10 was elevated after LPS treatment (Fig. 1b). However, despite slightly increased p38 activation, LPS-induced activation of IKKα/β and MAPKs, JNK and ERK, was normal in ZNRF1-deficient BMDMs and RAW264.7 macrophages (Fig. 1c and Supplementary Fig. 1c). In addition, ZNRF1-depleted macrophages exhibited a modest increase in the phosphorylation of IRF3 in response to LPS.

The cysteine residue at position 184 in the RING finger domain of ZNRF1 is reportedly critical for its E3 ubiquitin ligase activity^16^. To determine whether the E3 ubiquitin ligase activity of ZNRF1 is required for the modulation of TLR4-driven immune responses, we examined the induction of gene expression by LPS in *Znrf1*^Δ^ BMDMs reconstituted with either wild-type ZNRF1 or a catalytically inactive E3 ligase mutant ZNRF1(C184A). The LPS-induced *Tnf, Il6* and *Il1b* mRNA levels were increased in BMDMs after reconstitution with ZNRF1 but not in those reconstituted with the ZNRF1(C184A) mutant (Supplementary Fig. 1d). By contrast, *Il10* mRNA was downregulated in the ZNRF1-reconstituted BMDMs but not in those reconstituted with the ZNRF1(C184A) mutant, suggesting a requirement of its E3 ubiquitin ligase in the ZNRF1-mediated effects on the TLR4 response. Accordingly, elevated secretion of TNF and IL-6 levels and decreased IL-10 were found in supernatants of BMDMs reconstituted with ZNRF1 but not in those reconstituted with ZNRF1(C184A) mutant after stimulation with LPS (Fig. 1d). Taken together, these results indicate that ZNRF1 is involved in the regulation of cytokine production after TLR4 activation and the depletion of ZNRF1 impedes the coordination of cytokine synthesis in macrophages. These data confirm an important role for ZNRF1 in regulation of TLR4-induced pro-inflammatory cytokine production in macrophages, and this function requires the ubiquitin ligase activity ZNRF1, suggesting a novel mechanism for regulation of TLR4 signalling.

### *Znrf1* ^Δ^ mice are more resistant to septic shock

**Figure 2:**
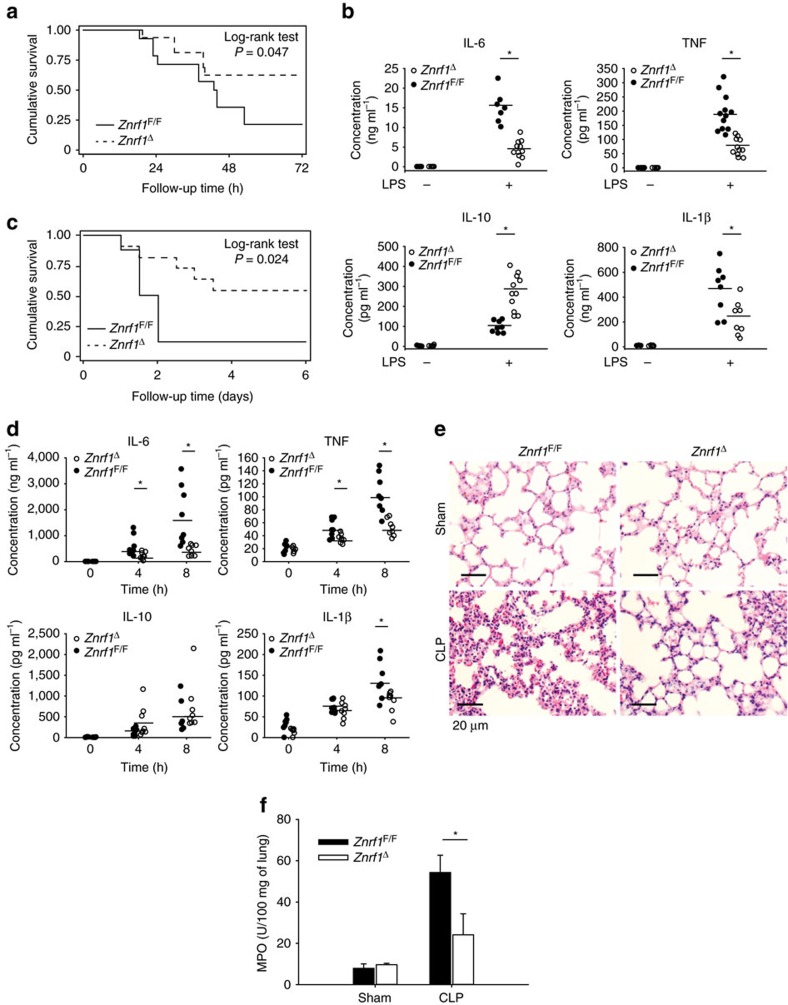
*Znrf1*^Δ^ mice are more resistant to LPS- and CLP-induced sepsis. (**a**) Survival of *Znrf1*^Δ^ (dotted line, *n*=11) and *Znrf1*^F/F^ mice (solid line, *n*=11) injected intraperitoneally with LPS (15 mg kg^−1^ of body weight). (**b**) ELISA analysis of the indicated cytokines in sera from *Znrf1*^Δ^ and *Znrf1*^F/F^ mice 6 h after LPS challenge. (**c**) Survival of *Znrf1*^Δ^ mice (dotted line, *n*=8) and *Znrf1*^F/F^ mice (solid line, *n*=8) after CLP challenge. (**d**) ELISA analysis of the indicated cytokines in sera from *Znrf1*^Δ^ and *Znrf1*^F/F^ mice collected 0, 4 and 8 h after the CLP procedure. (**e**) Haematoxylin and eosin staining of histological sections of lung tissues collected from *Znrf1*^Δ^ and *Znrf1*^F/F^ mice (lower panel) 8 h after CLP and from sham-operated control mice (upper panel). Objective magnification, × 20. Scale bar, 20 μm. (**f**) The MPO activity in lung tissues from *Znrf1*^Δ^ and *Znrf1*^F/F^ mice 8 h after CLP. *P<0.05 (Student's *t*-test). (Error bars, s.d.).

Sepsis is attributed to an exacerbated release of pro-inflammatory cytokines and results in high morbidity and mortality^17^. We assessed the physiological role of ZNRF1 in modulating inflammatory responses and septic shock *in vivo* by intraperitoneally injecting *Znrf1*^Δ^ and control mice with a lethal dose of LPS. After LPS challenge, more than 50% of the wild-type mice died within 48 h, whereas more than 60% of the *Znrf1*^Δ^ mice survived for up to 72 h (Fig. 2a). The serum concentrations of TNF, IL-6 and IL-1β induced by LPS were significantly lower, whereas the IL-10 level was higher, in *Znrf1*^Δ^ mice compared with their wild-type littermates (Fig. 2b). These results suggest that decreased plasma concentrations of pro-inflammatory cytokines contribute to the greater resistance of the *Znrf1*^Δ^ mice to endotoxin. In addition, we evaluated the effect of *Znrf1* deficiency on septic shock using the CLP model, a clinically relevant rodent model of polymicrobial sepsis. Consistent with the results of LPS-induced sepsis, the survival rate of *Znrf1*^Δ^ mice was higher than that of *Znrf1*^F/F^ mice (Fig. 2c). The serum levels of TNF, IL-6 and IL-1β after CLP were also lower in *Znrf1*^Δ^ mice than in *Znrf1*^F/F^ mice, whereas IL-10 expression was slightly higher in the *Znrf1*^Δ^ group (Fig. 2d). Other pro-inflammatory cytokines and chemokines, including IL-12p40, MCP-1, MIP-1α and CCL5, were also markedly decreased in CLP-treated *Znrf1*^Δ^ mice (Supplementary Fig. 4a). Histological analysis revealed significantly less inflammation and edema in lung tissue from *Znrf1*^Δ^ mice compared to *Znrf1*^F/F^ mice after CLP (Fig. 2e). In line with less pulmonary inflammation, myeloperoxidase activity, the neutrophil marker, was decreased in lung homogenates from CLP-challenged *Znrf1*^Δ^ mice (Fig. 2f). In addition, numbers of liver infiltrating macrophages was lower in CLP-treated *Znrf1*^Δ^ mice than control mice (Supplementary Fig. 4b). These results together demonstrate that ZNRF1 is a critical regulator of pro-inflammatory cytokine production during sepsis that has a significant impact on mortality.

### ZNRF1 regulates CAV1 protein turnover

**Figure 3:**
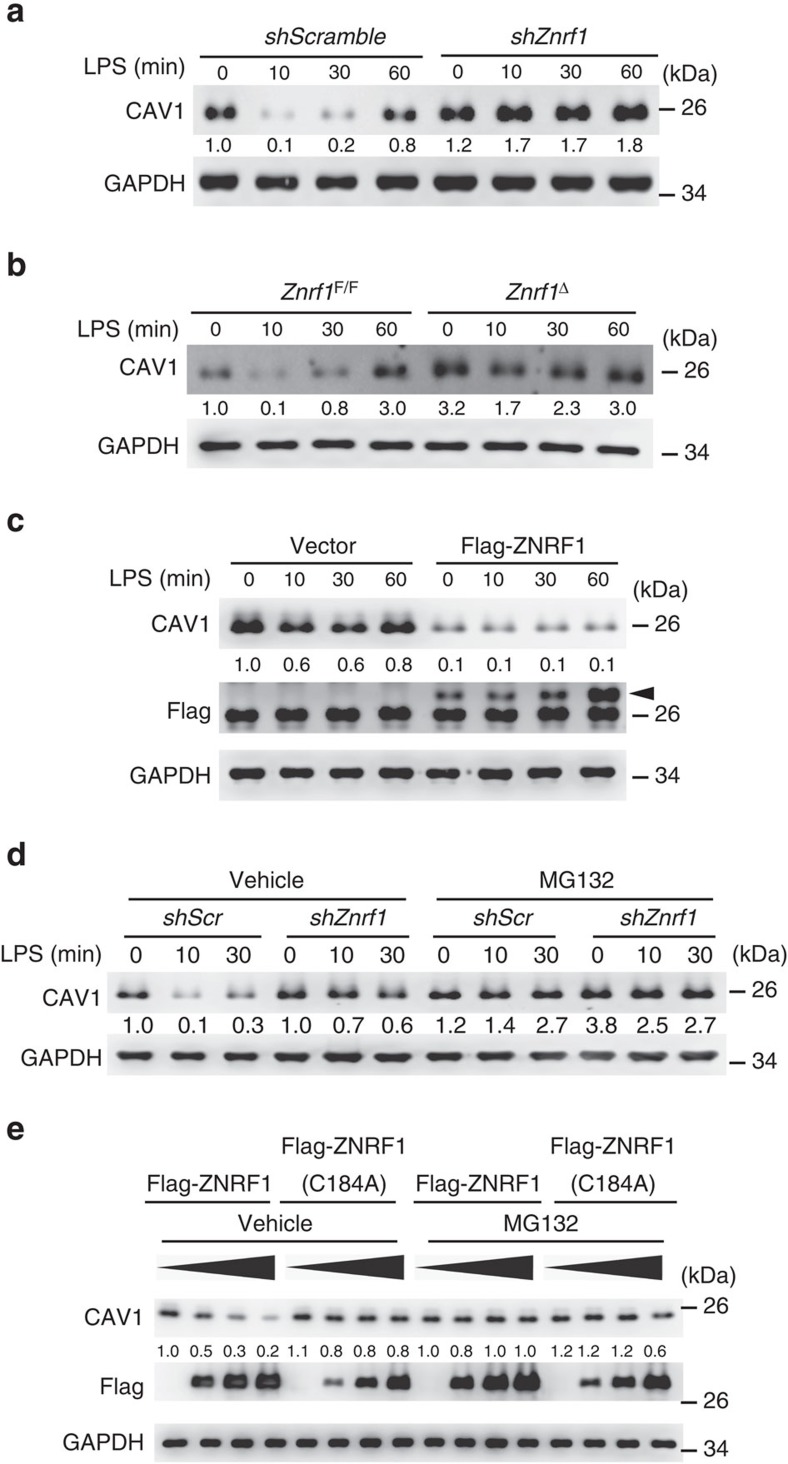
ZNRF1 regulates CAV1 protein stability. (**a**) Immunoblot analysis of CAV1 and GAPDH in lysates of control (scrambled shRNA) and ZNRF1-shRNA-expressing RAW264.7 cells stimulated with LPS (100 ng ml^−1^) for the indicated times. (**b**) Immunoblot analysis of CAV1 and GAPDH in lysates of *Znrf1*^F/F^ and *Znrf1*^Δ^ cells treated with LPS (100 ng ml^−1^) for the indicated times. (**c**) Immunoblot analysis of the indicated proteins in lysates of control (vector) and Flag-tagged ZNRF1-expressing RAW264.7 macrophages stimulated with LPS (100 ng ml^−1^). The arrow indicates Flag-ZNRF1. (**d**) Immunoblot analysis of CAV1 protein in lysates of scrambled control and *Znrf1*-knock-down RAW264.7 cells stimulated with 100 ng ml^−1^ LPS with or without pretreatment with MG132 (10 μM). (**e**) HEK293T cells were transfected with increasing amounts of Flag-tagged wild-type ZNRF1 or ZNRF1(C184A); 24 h after transfection, the cells were treated with MG132 (10 μM) for at least 6 h, and cell lysates were analysed by immunoblotting with the indicated antibodies. The intensities of the bands are expressed as fold increases compared to those of untreated control cells after normalization to GAPDH expression. The data are representative of three independent experiments performed in triplicate.

The pattern of cytokine production after LPS treatment in *Znrf1*^Δ^ BMDMs, that is, suppressed the induction of the pro-inflammatory cytokines TNF and IL-6 and elevated IL-10 production, was similar to that reported in CAV1-overexpressing macrophages^18^ and dendritic cells^19^. In addition, we consistently observed significantly higher CAV1 protein expression in liver from *Znrf1*^Δ^ mice after CLP challenge compared to littermate controls (Supplementary Fig. 4c,d). We thus examined CAV1 protein levels in both control and ZNRF1-depeleted macrophages. CAV1 protein levels were significantly increased in ZNRF1-silenced RAW264.7 cells, and this increase was even more prominent after LPS stimulation (Fig. 3a). We further found that CAV1 was increased at both the plasma membrane and the endosomes in *Znrf1*^Δ^ BMDMs (Supplementary Fig. 2c). This observation implied that ZNRF1 might regulate the protein expression of CAV1 after TLR4 activation. Similar results were observed in BMDMs from *Znrf1*^Δ^ mice (Fig. 3b). To verify the suppression of CAV1 protein expression by ZNRF1, we stably expressed Flag-tagged ZNRF1 and examined CAV1 expression. CAV1 protein was decreased when ZNRF1 was overexpressed in RAW264.7 cells (Fig. 3c), indicating that CAV1 protein level is controlled by ZNRF1. To confirm the effect of CAV1 on cytokine production following LPS stimulation, we generated stable RAW264.7 cells overexpressing CAV1. Similar to previous reports^20^, increased CAV1 level in macrophages attenuated the mRNA expression of pro-inflammatory cytokines but increased IL-10 mRNA (Supplementary Fig. 5a). However, activation of LPS-triggered MAPKs and IKKβ was not significantly affected by CAV1 expression (Supplementary Fig. 5b). These results strongly suggest that ZNRF1 regulates CAV1 expression and subsequently influences TLR4-induced cytokine production in macrophages.

The induction of CAV1 mRNA by LPS in either ZNRF1-depleted RAW264.7 cells or *Znrf1*^Δ^ BMDMs was not significantly different from that in the corresponding control cells, indicating that ZNRF1 regulates CAV1 expression by a posttranscriptional mechanism (Supplementary Fig. 6a). CAV1 protein degradation is reportedly dependent on both the ubiquitin/proteasomal and endolysosomal pathways^21^,^22^. Therefore, we further examined which pathway is involved in ZNRF1-regulated CAV1 protein stability. Pretreatment with MG132, a proteasome inhibitor, restored CAV1 levels in control cells but not ZNRF1-deficient cells (Fig. 3d). However, the lysosome inhibitors NH_4_Cl and chloroquine had a partial inhibitory effect on CAV1 degradation in both control and ZNRF1-silenced cells (Supplementary Fig. 6b). To determine whether ubiquitination is required for ZNRF1-controlled CAV1 protein expression, we expressed either wild-type ZNRF1 or the catalytically inactive E3 ligase mutant ZNRF1(C184A) in HEK293T cells. The protein levels of endogenous CAV1 were gradually reduced in cells expressing increasing amounts of ZNRF1 but not in cells expressing ZNRF1(C184A) (Fig. 3e). Similarly, the suppression effect by ZNRF1 was abolished in cells pretreated with MG132. ZNRF2 is a close structural homology of ZNRF1 (ref. 16). To examine the regulatory effect of ZNRF2 on CAV1 protein stability, we expressed ZNRF2 protein in HEK293T cells. The endogenous CAV1 protein levels were comparable in HEK293T cells with or without ZNRF2 expression (Supplementary Fig. 7a). In addition, CAV1 protein level and TLR4-driven signalling were not much significantly different between control and ZNRF2-depeleted RAW264.7 cells (Supplementary Fig. 7b), indicating that unlike ZNRF1, ZNRF2 does not modulate CAV1 turnover. Taken together, these data indicate that ZNRF1-mediated ubiquitination controls CAV1 degradation.

### ZNRF1 mediates the ubiquitination of CAV1

**Figure 4:**
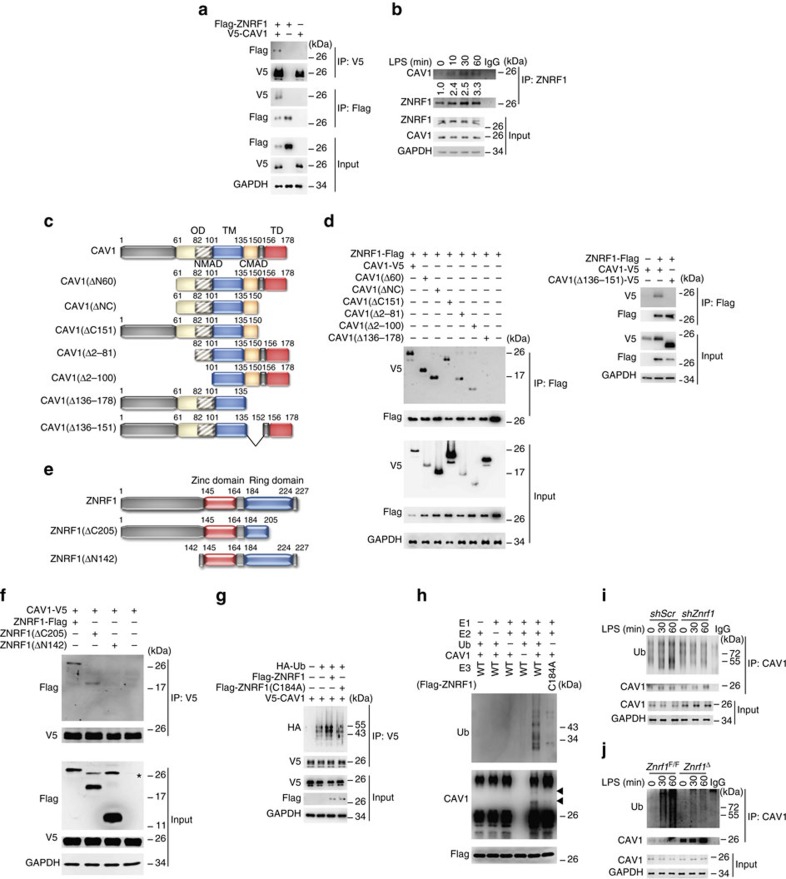
ZNRF1 interacts with CAV1 and promotes CAV1 ubiquitination in response to LPS. (**a**) HEK293T cells were co-transfected with V5-tagged CAV1 and Flag-tagged ZNRF1 as indicated. Interactions between ZNRF1 and CAV1 were detected by immunoprecipitation followed by immunoblot analysis with the indicated antibodies. (**b**) Immunoprecipitation of ZNRF1 from lysates of RAW264.7 cells treated with LPS (100 ng ml^−1^) for the indicated times, followed by immunoblot analysis with the indicated antibodies. The intensities of the bands are shown as fold increases compared to those of untreated cells after normalization to ZNRF1 level. (**c**) Schematic diagram of full-length CAV1 and six truncated forms of CAV1 with a C-terminal V5 tag. (**d**) HEK293T cells were co-transfected with Flag-tagged ZNRF1 and V5-tagged full-length or truncated CAV1 as indicated, and interactions between CAV1 and ZNRF1 were identified by immunoprecipitation and immunoblotting with the indicated antibodies. (**e**) Schematic diagram of full-length ZNRF1 and two truncated forms of ZNRF1 with a C-terminal Flag-tag. (**f**) HEK293T cells were co-transfected with V5-tagged CAV1 and Flag-tagged full-length or truncated form of ZNRF1 as indicated, and interactions between CAV1 and ZNRF1 were identified by immunoprecipitation and immunoblotting with the indicated antibodies. (**g**) HEK293T cells were co-transfected with Flag-tagged ZNRF1 or ZNRF1(C184A) mutant and V5-tagged CAV1 and HA-tagged ubiquitin. CAV1 ubiquitination was determined by immunoprecipitating V5-tagged CAV1 and subsequent immunoblotting with anti-HA. (**h**) *In vitro* ubiquitination assays were carried out with bacterially expressed His-CAV1, ZNRF1 or ZNRF1(C184A), purified ubiquitin catalytic components as indicated. The mixtures were then subjected to immunoblotting with the indicated antibodies. Arrows indicate Ubiquitin-conjugated CAV1. (**i**,**j**) RAW264.7 cells transduced with lentiviruses expressing *shScr* or *shZnrf1* and BMDMs from *Znrf1*^F/F^ and *Znrf1*^Δ^ mice were treated with LPS (100 ng ml^−1^) for the indicated times after MG132 pre-treatment. CAV1 ubiquitination was determined by immunoprecipitating CAV1 and subsequent immunoblotting with anti-ubiquitin antibody. The data are representative of three independent experiments performed in triplicate.

The specific E3 ubiquitin ligase responsible for CAV1 ubiquitination has not been identified. ZNRF1 is an E3 ubiquitin ligase and promotes AKT ubiquitination and degradation^23^. Thus, we speculated that ZNRF1 might directly ubiquitinate CAV1 and target it for degradation. We first examined whether ZNRF1 associates with CAV1 using reciprocal co-immunoprecipitation, which revealed a physical interaction between ZNRF1 and CAV1 transiently expressed in HEK293T cells (Fig. 4a). In addition, LPS stimulation in macrophages increased the interaction between endogenous ZNRF1 and CAV1 in a time-dependent manner (Fig. 4b). To map the domains responsible for the interaction between CAV1 and ZNRF1, we constructed six truncated forms of CAV1 (Fig. 4c) and two ZNRF1 deletion mutants (Fig. 4e). Co-IP experiments demonstrated that deletions of the C-terminal membrane attachment domain (C-MAD) only (CAV1(Δ136–151)) and C-MAD plus terminal domain (TD) in CAV1 (CAV1(Δ136–178)) resulted in their loss of the ability of CAV1 to bind to ZNRF1, whereas two CAV1 mutants (CAV1(ΔNC) and CAV1(Δ151)), lacking their extreme C-terminal region (aa 151–178), still retained the binding to ZNRF1 (Fig. 4d), indicating that CAV1 interacts with ZNRF1 via its C-MAD domain (aa 136–150). Deletion of the N-terminal domain (aa 1–142) in ZNRF1 (ZNRF1(ΔN142)) lost its ability to bind to CAV1 (Fig. 4f), suggesting that ZNRF1 interacts with CAV1 via its N-terminal domain.

To determine whether ZNRF1 directly catalyses CAV1 ubiquitination, we co-transfected HEK293 cells with wild-type ZNRF1 or ZNRF1(C184A) mutant, along with HA-tagged ubiquitin and determined the level of CAV1 ubiquitination. As expected, a significant increase in ubiquitinated CAV1 was detected in ZNRF1-overexpressing cells compared with cells transfected with vector control or the enzymatically inactive mutant ZNRF1(C184A) (Fig. 4g). To examine whether ZNRF1 directly ubiquitinates CAV1, we reconstituted purified recombinant His-tagged wild-type or mutant ZNRF1, CAV1 proteins, and other ubiquitination components, and carried out an *in vitro* ubiquitination assay. We observed CAV1 ubiquitination only when wild-type ZNRF1, but not its ligase inactive C184A mutant, was present (Fig. 4h). We then checked the effect of ZNRF1 deficiency on the ubiquitination of CAV1 in macrophages after treatment with LPS. LPS increased CAV1 ubiquitination in wild-type macrophages but had no significant effect on CAV1 ubiquitination in either ZNRF1-silenced RAW264.7 cells or *Znrf1*^Δ^ BMDMs (Fig. 4i,j). These results demonstrate that TLR4 activation triggers ZNRF1-dependent CAV1 ubiquitination.

**Figure 5:**
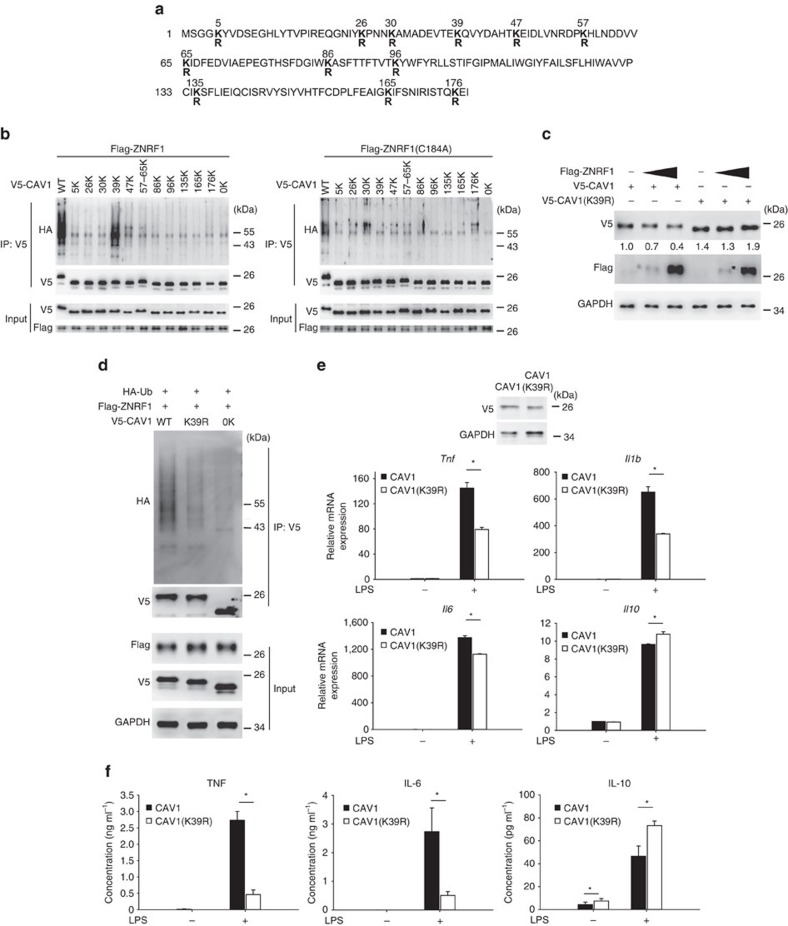
ZNRF1 mediates CAV1 polyubiquitination at lysine 39 and promote CAV1 degradation to modulate TLR4-mediated immune response. (**a**) Schematic diagram of mouse CAV1 sequence and its 12 lysine residues, which were mutated to arginines. (**b**) HEK293T cells were co-transfected with V5-tagged single-lysine CAV1 mutants and either Flag-ZNRF1 or ZNRF1(C184A) as indicated. CAV1 ubiquitination was determined by immunoprecipitating V5-CAV1 and immunoblotting with anti-HA. (**c**) Immunoblot analysis of V5-tagged CAV1 in lysates from HEK293T cells expressing increasing amounts of ZNRF1 and either wild-type CAV1 or the CAV1(K39R) mutant as indicated. The intensities of the bands are shown as fold increases compared to those of control cells after normalization to GAPDH expression. (**d**) Immunoprecipitation of V5-tagged CAV1 proteins in lysates from HEK29T cells expressing Flag-ZNRF1 and either V5-CAV1 or CAV1(K39R), followed by immunoblotting with anti-HA antibody. (**e**,**f**) *Cav1*^−/−^ BMDMs were reconstituted with either V5-CAV1 or CAV1(K39R) and treated with LPS (100 ng ml^−1^). (**e**) The mRNA expression levels of cytokines at 4 h post LPS were analysed by RT-qPCR, and (**f**) cytokine levels in supernatants were detected by ELISA at 12 h after LPS treatment. *P<0.05 (Student's *t*-test). The data are representative of three independent experiments performed in triplicate (error bars, s.d.).

Ubiquitination is a post-translational modification in which ubiquitin is covalently attached to a lysine residue on a target protein. The CAV1 protein contains 12 lysines (Fig. 5a), many of which are reported ubiquitin acceptor sites^21^,^24^. To determine which lysine site on CAV1 is the acceptor for the ZNRF1-mediated polyubiquitin chain, we systematically generated CAV1 mutants containing only a single lysine; all other lysines were mutated to arginine to allow conjugation of only one ubiquitin chain to CAV1. We also created a lysine-free CAV1 mutant (CAV1(0K)) by mutating all 12 lysines to arginines. The CAV1(39K) mutant, in which all lysines were mutated to arginine except lysine 39, exhibited a ubiquitination pattern similar to that of wild-type CAV1 when co-transfected with wild-type ZNRF1 into HEK293T cells (Fig. 5b). To further confirm that lysine 39 of CAV1 is critical for ZNRF1-mediated ubiquitination, we substituted lysine 39 of CAV1 with arginine (CAV1(K39R)). CAV1(K39R) conferred resistance to ZNRF1-mediated degradation compared to wild-type CAV1 when transiently expressed in HEK293T cells (Fig. 5c). Congruently, CAV1(K39R) expression was associated with a significant decrease in ZNRF1-mediated polyubiquitination (Fig. 5d). To determine if ZNRF1-mediated CAV1 ubiquitination is involved in LPS-induced cytokine production, we reconstituted *Cav1*^−/−^ BMDMs with wild-type CAV1 or CAV1(K39R) mutant. The reconstitution of *Cav1*^−/−^ BMDMs with CAV1(K39R) was associated with lower LPS-stimulated induction of TNF, IL-1β and IL-6 mRNA but higher IL-10 mRNA production compared with wild-type CAV1 (Fig. 5e). Corresponding to mRNA expression, cytokine production of TNF and IL-6 were decreased and IL-10 was enhanced after TLR4 activation in cells reconstituted with CAV1(K39R) (Fig. 5f). In conclusion, the E3 ubiquitin ligase activity of ZNRF1 regulates TLR4-dependent immune responses by controlling the stability of CAV1.

### ZNRF1 regulates inflammation via the Akt–GSK3β pathway

**Figure 6:**
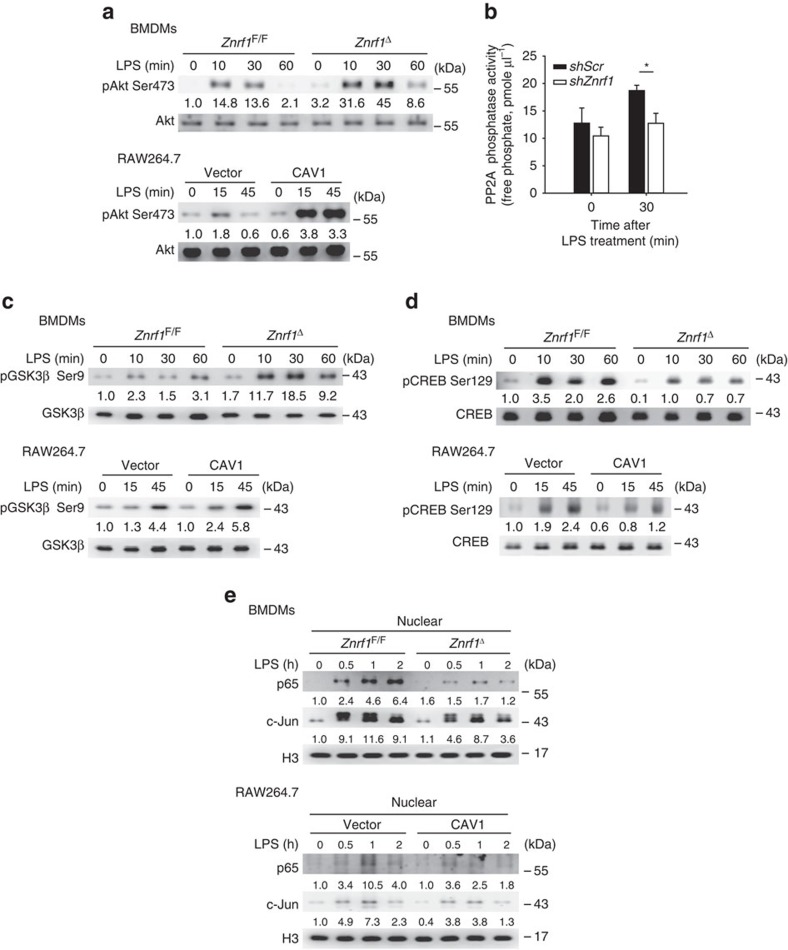
Akt–GSK3β–CREB signalling is altered in ZNRF1-deficient and CAV1-expressing macrophages in response to LPS. (**a**,**c**,**d**) Immunoblot analysis of the phosphorylation of Akt (Ser473), GSK3β (Ser9) and CREB (Ser129) as well as total Akt, GSK3β, and CREB in lysates of *Znrf1*^F/F^ and *Znrf1*^Δ^ BMDMs and vector- and CAV1-expressing RAW264.7 cells stimulated with LPS (100 ng ml^−1^) for the indicated times. (**b**) RAW264.7 macrophages infected with lentiviruses expressing *shScr* or *shZnrf1* were treated with LPS (100 ng ml^−1^) for 30 min, and cell lysates were prepared and PP2A catalytic subunit was immunoprecipitated followed by PP2A phosphatase activity analysis. (**e**) Immunoblot analysis of NF-κB p65 and c-Jun in nuclear extracts isolated from BMDMs and RAW264.7 cells described above. Histone H3 served as a marker for the nuclear fraction. The intensities of the bands are shown as fold increases compared to those of untreated control cells after normalization to their unphosphorylated forms or H3 expression. *P<0.001 (Student's *t*-test). The data are representative of three independent experiments performed in triplicate (error bars, s.d.).

It was shown that CAV1 overexpression enhanced Akt activity, followed by decreased activation of the downstream kinase GSK3β via phosphorylation at serine 9 (refs 25, 26). The suppression of GSK3β activation was reported to decrease the production of pro-inflammatory cytokines and increase IL-10 expression in LPS-stimulated immune cells^27^,^28^. Therefore, we examined whether the Akt–GSK3β signalling pathway is responsible for the dysregulated production of LPS-induced pro-inflammatory and anti-inflammatory cytokines in ZNRF1-deficient BMDMs. After LPS stimulation, phosphorylation of Akt1 at serine 473, which represents the activated form of Akt1, was enhanced in both *Znrf1*^Δ^ BMDMs and CAV1-overexpressing RAW264.7 macrophages (Fig. 6a). The protein phosphatase 2A (PP2A), a known dephosphorylation enzyme for Akt, was previously reported to be blocked by CAV1 (ref. 25). To determine whether increased Akt activation in LPS-treated ZNRF1-depleted cells was due to decreased PP2A activity, we monitored the PP2A activity in both *shZnrf1* and *shScr* cells. PP2A phosphatase activity was indeed impaired in ZNRF1 depletion cells upon TLR4 engagement (Fig. 6b). In addition, phosphorylation of GSK3β at serine 9 (inactive form of GSK3β) was elevated in both *Znrf1*^Δ^ BMDMs and CAV1-overexpressing RAW264.7 cells, indicating that increasing CAV1 expression suppresses GSK3β activity by enhancing Akt activation in TLR4-activated macrophages (Fig. 6c). GSK3β phosphorylates the transcription factor CREB at serine 129, resulting in a loss of the DNA-binding activity of CREB^29^,^30^. CREB was shown to be a critical positive regulator of IL-10 transcription, and inhibiting GSK3β activation enhanced CREB activity and, consequently, increased IL-10 mRNA expression^31^. Thus, we assessed whether Akt–GSK3β signalling upregulated LPS-triggered IL-10 production through CREB activation in *Znrf1*^Δ^ BMDMs. Indeed, we observed that phosphorylation of CREB at serine 129 was reduced in *Znrf1*^Δ^ BMDMs and CAV1-overexpressing RAW264.7 macrophages after LPS stimulation, indicating an increase in CREB transactivational activity (Fig. 6d). It has been demonstrated that GSK3β negatively regulates NF-κB activation by inhibiting p65 nuclear translocation^32^,^33^. Therefore, the blunted cytokine production in LPS-treated ZNRF1-deficient cells may have been due to reduced nuclear translocation of NF-κB via the Akt–GSK3β signalling pathway. As expected, nuclear translocation of NF-κB subunit p65 was markedly decreased in both *Znrf1*^Δ^ BMDMs and CAV1-upregulated RAW264.7 macrophages compared with control macrophages (Fig. 6e). However, there was little decrease in TLR4-activated c-Jun nuclear localization. These data collectively demonstrate that ZNRF1 modulates the induction of TLR4-induced pro- and anti-inflammatory cytokines through the regulation of CAV1 expression and the Akt–GSK3β signalling pathway.

### ZNRF1-modulated inflammatory responses require CAV1

**Figure 7:**
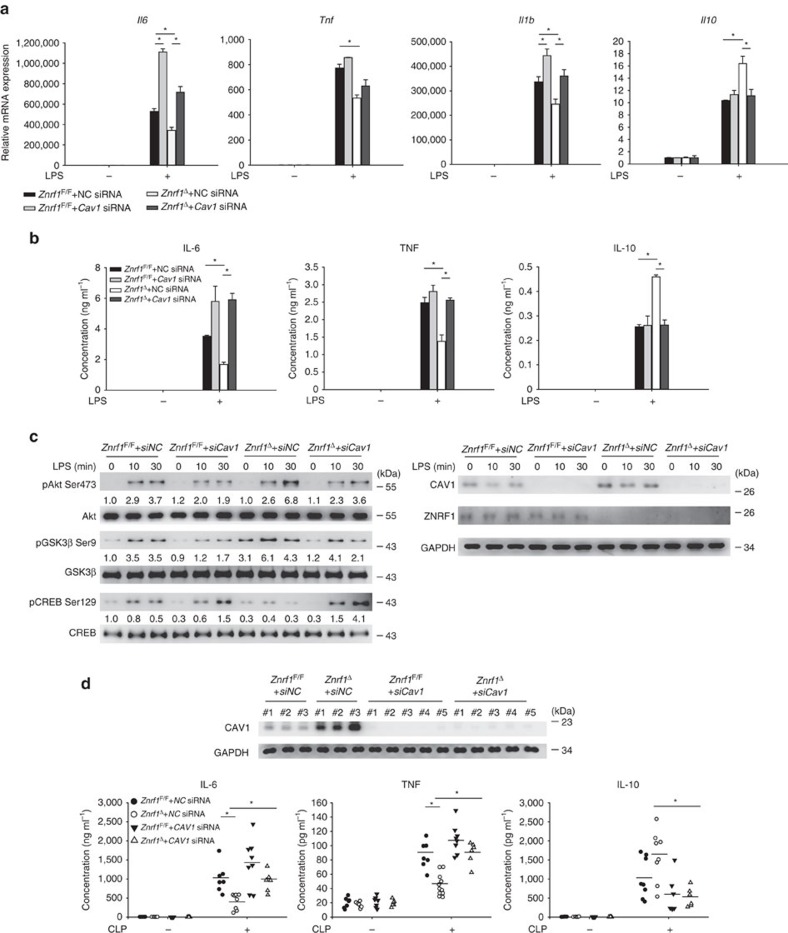
ZNRF1-mediated TLR4- and CLP-triggered inflammatory responses depend on CAV1. (**a**–**c**) *Znrf1*^F/F^ or *Znrf1*^Δ^ BMDMs electroporated with either control (*NC)* or *Cav1* siRNA were treated with LPS (100 ng ml^−1^). (**a**) The expression of the indicated mRNAs in BMDMs after stimulation with LPS for 4 h was analysed by RT–qPCR. (**b**) The production of cytokines in culture supernatants of BMDMs after treatment with LPS for 8 h was determined by ELISA. (**c**) Phosphorylation of Akt, GSK3β, and CREB as well as the indicated proteins in cell lysates were analysed by immunoblotting with the indicated antibodies. The intensities of the bands are shown as fold increases compared to those of untreated control cells after normalization to their unphosphorylated forms. The data are representative of three independent experiments performed in triplicate (error bars, s.d.). (**d**) *Znrf1*^F/F^ or *Znrf1*^Δ^ mice were injected intravenously with either control (*NC)* or *Cav1* siRNA. After 36 h, mice were subjected to CLP, and blood was collected 8 h post CLP. The indicated cytokines in sera were determined by ELISA (bottom), and CAV1 protein level in peripheral blood leukocytes were analysed by immunoblotting (top). *P<0.05 (Student's *t*-test).

To further confirm ZNRF1-mediated TLR4-activated signalling and cytokine production through CAV1, we depleted CAV1 expression by siRNA in both *Znrf1*^F/F^ and *Znrf1*^Δ^ BMDMs. When CAV1 was downregulated in *Znrf1*^Δ^ BMDMs, increased *Il10* and decreased *Il1b*, *Il6* and *Tnf* expression were almost diminished after TLR4 engagement compared to control (negative control (*NC*)) siRNA (Fig. 7a). Accordingly, *Znrf1*^Δ^ BMDMs with CAV1 depletion had less IL-10, but higher proinflammatory cytokines production in response to LPS (Fig. 7b). Furthermore, phosphorylation of Akt1 at Ser 473 and GSK3β at Ser 9 was severely reduced, whereas CREB phosphorylation at Ser 129 was elevated in LPS-activated *Znrf1*^Δ^ BMDMs transfected with CAV1 siRNA, but not in that with control siRNA (Fig. 7c). To further confirm the physiological effect of the ZNRF1–CAV1 axis in modulating inflammatory responses *in vivo*, we systemically depleted CAV1 in *Znrf1*^F/F^ and *Znrf1*^Δ^ mice by intravenous administration of siRNA. Consistent with the result in BMDMs, depletion of CAV1 in *Znrf1*^Δ^ mice substantially increased plasma IL-6 and TNF, and decreased IL-10 level after CLP challenge (Fig. 7d). Hence, ZNRF1 regulated TLR4-triggered Akt–GSK3β signalling and inflammation through controlling CAV1 protein level.

## Discussion

Inflammatory response is quickly induced by the innate immune system, mainly TLRs, upon infection to combat pathogens, but this process need to be tightly controlled^34^. TLR4 plays a central role in the recognition of both Gram-negative and Gram-positive bacteria^1^. However, despite extensive investigation, the regulation of TLR4-mediated inflammatory responses remains fragmentary. In this study, we identified an E3 ubiquitin ligase, ZNRF1, that modulates CAV1 ubiquitination and degradation to regulate TLR4-activated immune responses *in vitro* and *in vivo*. ZNRF1 deficiency resulted in decreased CAV1 ubiquitination and protein turnover, which subsequently inhibited TLR4-initiated inflammatory milieu. Mechanistically, ZNRF1-modulated CAV1 expression regulates Akt–GSK3β signalling, whose downstream targets inhibit inflammatory responses by suppressing the induction of pro-inflammatory cytokines and enhancing IL-10 production. Our findings thus demonstrate that ZNRF1 is a novel regulator of TLR4-induced inflammation through controlling CAV1 protein level.

CAV1 is a major protein component of a special subset of plasma membrane microdomains known as caveolae, and CAV1 has a variety of biological functions, including membrane receptor trafficking, tumour growth and migration, and lipid transport^35^,^36^. The functional roles of CAV1 in innate immunity and inflammation remain controversial and appear to depend on cell type. The depletion of CAV1 in murine macrophages lead to higher production of pro-inflammatory cytokines (TNF and IL-6) and lower anti-inflammatory cytokine IL-10 expression upon LPS challenge^37^, indicating an anti-inflammatory role of CAV1. However, CAV1-null mice are more resistant to LPS challenge due to a reduction in lung inflammation^38^,^39^. These findings have been ascribed to the direct or indirect downregulation of TLR4-induced NF-κB activation by CAV1 deletion^37^,^39^, which suggests that CAV1 positively regulates TLR4 signalling in pulmonary endothelial cells. However, recent studies have demonstrated that *Cav1*^−/−^ mice are more susceptible to infection with various pathogenic bacteria or to CLP challenge^40^,^41^ due to an enhanced inflammatory response, revealing a protective role of CAV1 in bacteria-induced sepsis. Because myeloid cells, including macrophages, are predominately responsible for cytokine production during sepsis^10^, the function of myeloid CAV1 during sepsis remains elusive. Thus, myeloid cell-specific CAV1-deficient mice are needed to address this issue. Our results showed that ZNRF1 negatively modulates CAV1 protein expression. Both ZNRF1-deficient and CAV1-overexpressing macrophages exhibited decreased LPS-induced pro-inflammatory cytokine expression but augmented IL-10 induction in our and other studies^20^,^38^. This phenotype was vanished when CAV1 was further knocked down in ZNRF1-deficient cells. The major hallmark of sepsis is exaggerated inflammatory responses^42^. We demonstrated that myeloid cell-specific ZNRF1 deletion protects mice from both LPS- and CLP-induced septic death, and depletion of CAV1 reversed the attenuated inflammatory response and possible survival in *Znrf1*^Δ^ mice after CLP, indicating a pro-inflammatory function of ZNRF1. Our results also suggest that the anti-inflammatory role of CAV1 in bacterial- and CLP-induced sepsis is most likely dependent on its dysregulation of cytokine production in myeloid cells.

Our data revealed that ZNRF1 controls CAV1 protein expression via an ubiquitin-dependent degradation mechanism, and does not share this function with its closest family member, ZNRF2. Ubiquitination is crucial for maintaining CAV1 protein levels, although an endolysosomally mediated pathway is also involved^21^,^22^,^43^. However, no E3 ubiquitin ligases responsible for CAV1 ubiquitination have yet been characterized. To our knowledge, this is the first report describing an E3 ubiquitin ligase that regulates CAV1 protein ubiquitination and degradation.

ZNRF1 was recently shown to target and degrade Akt through the ubiquitin–proteasome system in neuronal cells^23^. However, we observed no change in Akt protein level but rather hyper-phosphorylation of Akt1 in *Znrf1*^Δ^ BMDMs after TLR4 activation. Our findings demonstrate that in *Znrf1*^Δ^ BMDMs, TLR4 activation causes elevated phosphorylation of Akt, followed by GSK3β inactivation, eventually resulting in reduced NF-κB nuclear translocation and enhanced CREB transcriptional activity. A similar effect was observed in CAV1-overexpressing RAW264.7 macrophages after LPS stimulation, suggesting a new regulatory role for ZNRF1–CAV1 signalling in TLR4-induced immune responses. Despite TLR4-induced IL-10 expression was previously reported to be dependent on type I interferon-IL-27 signalling^44^,^45^, the Akt–GSK3β–CREB pathway was also critical for the regulation of IL-10 production during TLR4 activation^27^,^30^. In addition, Akt was previously shown to suppress GSK3β activation, leading to impaired NF-κB activation^46^. Consistent with these published findings, elevated CREB activation and attenuated NF-κB p65 nuclear translocation were observed in LPS-challenged ZNRF1-deficient and CAV1-expressing macrophages in the present work, suggesting that ZNRF1-CAV1 mainly functions to regulate Akt–GSK3β–CREB/p65 signalling. It was previously reported that the overexpression of CAV1 suppresses p65 acetylation, thereby inhibiting NF-κB activity^47^. However, we did not detect diminished p65 acetylation in *Znrf1*^Δ^ BMDMs after LPS challenge (data not shown). Nevertheless, we are still unable to exclude the possibility that ZNRF1 modulates NF-κB activity through other mechanisms to control the induction of pro-inflammatory cytokines.

Our data suggest that upregulation of CAV1 caused by ZNRF1 depletion augments TLR4-induced Akt activation through inhibition of PP2A phosphatase activity. Numerous studies have previously demonstrated a suppressive effect of CAV1 on PP2A activity^25^,^48^,^49^. However, a class IA PI3Kδ was previously shown to recruit TLR4 to the plasma membrane, resulting in Akt activation and TLR4 internalization^50^. We observed no difference in LPS-induced TLR4 internalization between control and ZNRF1-deficient macrophages (data not shown). A recent report demonstrated that Rab8a, a small GTPase, is enriched in the dorsal ruffles of TLR4-activated macrophages, where it recruits and activates a class IB PI3Kγ (ref. 51). The Rab8a–PI3Kγ complex then activates downstream Akt activity, leading to the inhibition of inflammatory responses by attenuating the production of pro-inflammatory cytokines and enhancing the release of IL-10, but does not affect TLR4 internalization. The effect of Rab8a–PI3Kγ–Akt signalling on TLR4-mediated immune responses is very similar to that in ZNRF1-deficient macrophages. Thus, we cannot exclude the possibility that ZNRF1–CAV1 signalling regulates TLR4-induced inflammatory responses through the modulation of PI3Kγ recruitment and activation, although this hypothesis remains to be verified. In addition, the upregulation of CAV1 in macrophages appears to affect the TLR4–MyD88 signalling pathway only. We also observed increased activation of IRF3, which is regulated by endosomal TLR4–TRIF signalling, in LPS-treated *Znrf1*^Δ^ BMDMs. The role of ZNRF1 in endosomal TLR4 signalling and the underlying mechanism await further elucidation.

The activation of macrophages by the stimulation of the TLR4 ligand leads to the production of several inflammatory mediators, including TNF, IL-1β and IL-6, that contribute to the inflammatory response to defend pathogenic infection, but also associate with the severity of sepsis. Our data demonstrated that LPS induces a transient association between CAV1 and ZNRF1 as well as a decrease in CAV1 protein levels through the ubiquitin/proteasomal pathway. These effects of LPS allow the innate response to quickly shift towards pro-inflammatory cytokine/chemokine production, which is needed to constrain pathogens during the early phase of host infection. LPS was known to induce CAV1 expression^52^. Thereby, upregulated CAV1 level might contribute to the suppression of inflammation at later phase of infection. CAV1 levels are reportedly upregulated in aged and senescent cells and animals^53^. Therefore, it may be worthwhile to investigate age-related defects in innate defenses against pathogen infection in association with the expression of CAV1 and ZNRF1. In summary, we determined that ZNRF1 regulates CAV1 turnover and further modulates inflammatory responses in response to endotoxin, identifying the ZNRF1–CAV1 axis as a new and non-redundant mechanism for controlling inflammation (Supplementary Fig. 8). Because excessive CAV1 expression contributes to a variety of human diseases, including cancer metastasis, atherosclerosis, and diabetes, our findings may also indicate a novel therapeutic target for the treatment of these CAV1-related diseases.

## Methods

### Plasmids

Full-length ZNRF1 and ZNRF2 cDNAs were amplified by PCR from a mixture of cDNAs derived from C57BL/6 mouse livers and cloned into pcDNA3 (Invitrogen, Carlsbad, CA) containing a C-terminal Flag epitope tag. A plasmid encoding Flag-tagged ZNRF1(C184A) mutant was generated by site-directed mutagenesis using the QuickChange II Site-Directed Mutagenesis Kit (Stratagene, La Jolla, CA). Flag-ZNRF1 and Flag-ZNRF1(C184A) were subsequently cloned into the pLKO-AS2 lentivirus-based vector (National RNAi Core Facility, Academia Sinica, Taiwan) for lentivirus production. CAV1 cDNA was PCR amplified and cloned into a C-terminally V5-tagged pLKO-AS2 lentiviral vector. The CAV1 and ZNRF1 deletion mutants were generated by PCR using full-length CAV1 and ZNRF1 as templates. All mutations were confirmed by DNA sequencing. The pcDNA-HA-Ubiquitin plasmid was obtained from Addgene (Cambridge, MA).

### Reagents and antibodies

Anti-ZNRF1 polyclonal antibody was generated in rabbits immunized with a synthetic peptide corresponding to amino acids 71–86 (amino-acid sequence LYTPASRGTGDSERAP) of human ZNRF1 conjugated to keyhole limpet hemocyanin (KLH). Rabbit antisera were collected and further purified by Protein A affinity chromatography. Antibodies against p65 (#8642, 1:1,000), GAPDH (#5174, 1:1,000), H3 (#4499, 1:1,000), phospho-IKKα/β (#2697, 1:1,000), phospho-p38 (#9211, 1:1,000), phospho-JNK1/2 (#9251, 1:1,000), phospho-ERK1/2 (#4370, 1:1,000), p38 (#8690, 1:1,000), JNK1/2 (#554285, 1:1,000), ERK1/2 (#9102, 1:1,000), CAV1 (#3267, 1:1,000), phospho-GSK3β (Ser9) (#5558, 1:1,000), phospho-AKT (Ser473) (#4060, 1:1,000), GSK3β (#12456, 1:1,000), and AKT (#1691, 1:1,000) were obtained from Cell Signaling Technology (Danvers, MA). The anti-c-Jun (sc7694, 1:1,000), IKKα/β (sc7607, 1:1,000), and phospho-CREB (Ser129) (sc101662, 1:2,000) antibodies were from Santa Cruz Biotechnology (Santa Cruz, CA) and anti-β-actin antibody (MAB1501, 1:5,000) was from Millipore (Billerica, MA). The anti-ubiquitin (MMS258R, 1:1,000) and ZNRF2 (ab156011, 1:1,000) antibodies were purchased from Covance (Princeton, NJ) and Abcam (Cambridge, UK), respectively. Antibodies against V5 (A190-120P, 1:5,000) and HA (A190-108P, 1:2,000) and anti-Flag-conjugated Sepharose (S190-101, 2.5 μg) were provided by Bethyl Laboratories (Montgomery, TX). The anti-Flag (637301, 1:5,000) and anti-CD68 (25747-1-AP, 1:100) antibodies were obtained from BioLegend (San Diego, CA) and Proteintech (Chicago, IL). High-molecular-weight (HMW) polyinosinic-polycytidylic acid (poly(I:C)) and macrophage colony-stimulating factor (M-CSF) were from InvivoGen (San Diego, CA) and Peprotech (Rocky Hill, NJ), respectively. All other chemicals, including LPS (*Escherichia coli* 0111:B4), were purchased from Sigma-Aldrich (St Louis, MO).

### Mice

*Znrf1*^F/F^ mice were generated via two homologous recombination events in embryonic stem cells (ES cells) to insert two *loxP* sites into the first and third introns of the *Znrf1* gene because of the long second intron. To generate hematopoietic cell-specific *Znrf1*-deletion mice, we crossed *Znrf1*^F/F^ mice with interferon-inducible Mx1 promoter-driven Cre recombinase transgenic mice^54^. To delete *Znrf1* in *Znrf1*^F/F^*:Mx1-Cre* mice, 3- to 4-week-old mice were injected intraperitoneally with 10 μg g^−1^ poly(I:C) twice every other day; the mice were used in experiments 2 weeks later. These mice are referred to as *Znrf1*^Δ^ mice. C57BL/6 mice were obtained from the Animal Center of the National Taiwan University Medical College. *Znrf1*^F/F^ mice were backcrossed with C57BL/6 mice for at least nine generations and housed in a specific pathogen-free animal facility. All animal experiments were conducted in accordance with animal welfare guidelines and were approved by the Institutional Animal Care and Use Committee (IACUC) of the College of Medicine, National Taiwan University (approval no. 20120220).

### Cell cultures and BMDM preparation

Murine macrophage-like RAW264.7 cells and human promonocytic leukemia cells THP-1 were cultured in RPMI 1640 (Hyclone, Logan, UT) supplemented with 10% (vol/vol) heat-inactivated fetal bovine serum (FBS) and 100 U ml^−1^ penicillin/streptomycin. THP-1 cells were differentiated into macrophages by treating cells with 25 ng ml^−1^ of phorbol 12-myristate 13-acetate for 4 h, followed by overnight incubation in fresh medium before experiments. Human embryonic kidney HEK293T cells were cultured in DMEM containing 10% FBS and 100 U ml^−1^ penicillin/streptomycin. Bone marrows from 6- to 8-week-old mice were used to prepare BMDMs as previously described^55^. Briefly, femurs and tibia bones were collected from mice, and bone marrow was flushed out with DMEM medium using a 25-gauge syringe. The bone marrow cells were collected and cultured in high-glucose DMEM medium supplemented with 20% L929 cell-conditioned medium for 7 days to differentiate into macrophages. BMDMs were collected and cultured in DMEM containing M-CSF (10 ng ml^−1^) for further experiments. Mycoplasma contamination in cell cultures was routinely detected by PCR using the primes, forward: 5′-GGGAGCAAACACGATAGATACCCT-3′ and reverse: 5′-TGCACCATCTGTCACTCTGTTAACCTC-3′, as suggested previously^56^.

### mRNA purification and quantitative RT–PCR

Total cellular RNA was isolated using the RNeasy Mini Kit (Qiagen, Valencia, CA) and used to synthesize cDNA with the RevertAid H Minus First Strand cDNA Synthesis Kit (Thermo Scientific, Rockford, IL) according to the manufacturer's instructions. The cDNA amounts were measured using real-time quantitative PCR (qPCR) with Maxima SYBR Green/Fluorescein qPCR Master Mix (Thermo Scientific, Rockford, IL) according to the manufacturer's recommendations. All qPCR values were normalized to cyclophilin mRNA levels to obtain the relative values. All experiments were performed in triplicate. The primer sequences are shown in Supplementary Table 1.

### shRNA-mediated gene silencing and lentiviral infection

Lentiviral shRNA constructs encoding short hairpin RNAs (shRNAs) against mouse *Znrf1* in pLKO-puro vectors (pLKO-shZnrf1) were obtained from the National RNAi Core Facility Platform at the Institute of Molecular Biology/Genomic Research Center, Academia Sinica, Taiwan. The target sequences were 5′-CATGGTTTGAAGTGAACAGAT-3′ for *Znrf1* shRNA 1, 5′-GAGATGGAAATGCACTTTATA-3′ for *Znrf1* shRNA2, and 5′-GATGAAATGGATTTGCATCTT-3′ for *Znrf2* shRNA. HEK293T cells were transfected with pLKO-shRNA constructs and the packaging plasmids pMD.G and pCMVR8.91 using Turbofect (Fermentas, Schwerte, Germany) according to the manufacturer's directions. Culture medium containing the lentivirus was collected 48 and 72 h after transfection. RAW264.7 and THP-1 cells were infected overnight with lentiviruses in the presence of 8 μg ml^−1^ polybrene (Sigma-Aldrich, St Louis, MO) and cultured in fresh medium for another 24 h. The infected cells were selected in medium containing 5 μg ml^−1^ puromycin until the uninfected cells were completely killed. The stable colonies were pooled together for further experiments. Bone marrow cells were transduced with shRNA-encoding lentiviruses on days one and two of differentiation into macrophages as described previously^57^.

### siRNA duplexes and electroporation

Predesigned siRNA against mouse *Cav1* (5′-GCAAAUUUAUGAAUGGUUUdTdT-3′), and the negative control siRNA (5′-GAUCAUACGUGCGAUCAGAdTdT-3′) were obtained from Sigma-Aldrich (St Louis, MO). For electroporation, BMDMs were electroporated with 100 nM siRNA duplexes using the Neon transfection system (Invitrogen) according to the manufacturer's instructions. Briefly, 5 × 10^5^ cells were electroporated with two 30 ms pulses at 1,500 V, and cells were cultured for another 48 h before further experiments.

### ELISA and myeloperoxidase activity assay

The levels of cytokines and chemokines in sera and culture supernatants were measured using DuoSet enzyme-linked immunosorbent assay (ELISA) systems (R&D Systems, Minneapolis, MN) according to the manufacturer's recommendations. The myeloperoxidase (MPO) activity in mouse lung homogenates was determined using the MPO colorimetric assay (BioVision, Milpitas, CA) following the manufacturer's instructions.

### Immunoprecipitation and immunoblotting

Whole cells were collected and lysed in ice-cold lysis buffer containing 150 mM NaCl, 20 mM Tris-HCl pH 7.4, 0.5% Triton X-100 and protease inhibitor cocktail (Roche, Basel, Switzerland) and homogenized by sonication in 1.5-ml microfuge tubes on ice for 30 s. Protein concentrations were determined using the Bradford assay (Bio-Rad, Hercules, CA). Cellular extracts (200 μg) were incubated with the indicated antibody overnight, followed by a 1-h incubation with Protein A/G beads or antibody-conjugated beads overnight at 4 °C. The immunocomplexes were separated by SDS-polyacrylamide gel electrophoresis and transferred to nitrocellulose membranes. The membranes were then incubated with the indicated primary antibody followed by an HRP-conjugated secondary antibody. Immunoreactive bands were detected using Western Lighting Plus-ECL (PerkinElmer, Waltham, MA).

### *In vitro* ubiquitination assay

Recombinant His-ZNRF1-Flag, His-ZNRF1(C184A)-Flag, and His-CAV1-V5 proteins were expressed in *E. coli* BL21, and purified using Ni-nitrilotriacetic acid (Ni-NTA) resins (Macherey-Nagel, Düren, Germany) following manufacturer's recommendations. Specific E1 and E2 were obtained from Enzo Life Sciences (Farmingdale, NY). *In vitro* ubiquitination assays were performed by incubating 500 ng His-tagged ZNRF1-Flag or ZNRF1(C184A)-Flag, and 500 ng His-CAV1-V5 together with 1 μg ubiquitin (Boston Biochem, Cambridge, MA), 100 ng His–Ube1, and 150 ng His–UbcH5c in the reaction buffer (25 mM Tris–HCl pH 8.0, 100 mM NaCl, 1 mM DTT, 1 mM MgCl_2_, 100 μM  ZnSO_4_, 2 mM ATP), at 30 °C for 3 h. The reactions were terminated by adding lysis buffer (150 mM NaCl, 20 mM Tris–HCl pH 7.4, 1% SDS) and subjected to immunoblotting.

### PP2A activity measurement

Cells were lysed in the lysis buffer containing 50 nM Tris–HCl pH7.4, 150 mM NaCl, 1 mM EDTA, 1% Triton-X100 and protease inhibitor. Cell lysates were immunoprecipitated with antibody against PP2A catalytic subunit for 2 h. The immunoprecipitants were subjected to PP2A phosphatase activity assay using the PP2A Immunoprecipitation Phosphatase assay kit (Millipore, Billerica, MA) according to the manufacturer's instructions.

### *In vivo* administration of siRNAs

siRNA (Ambion *In Vivo* siRNA, Thermo Fisher Scientific) was delivered *in vivo* using *in vivo*-jetPEI (Polyplus-transfection, Illkirch, France) according to the manufacturer's protocols. In brief, 40 μg siRNA duplex was mixed with 6.4 μl *in vivo*-jetPEI in a final volume of 200 μl of 10% glucose. The mixture was incubated for 15 min at room temperature and was delivered to the mice by intravenous injection through the femoral vein.

### Animal models of endotoxemia and polymicrobial sepsis

To induce endotoxemia, age- and weight-matched male *Znrf1*^F/F^ and *Znrf1*^Δ^ mice were injected intraperitoneally with bacterial endotoxin (LPS; 15 mg kg^−1^). Blood samples were collected 6 h after LPS challenge, and serum cytokines were measured using ELISA. Polymicrobial sepsis was induced by CLP as previously described^55^. Briefly, mice were anesthetized and maintained with isoflurane, and a 2-cm midline laparotomy was performed to allow the exposure of the cecum and the adjacent intestine. A 3.0 silk suture was used to tightly ligate the cecum at its base below the ileocecal valve, and the cecum was punctured once with a 21-gauge needle. The cecum was then gently returned to the peritoneal cavity. The laparotomy was closed with a 5.0 silk suture. All mice were subcutaneously injected with sterile saline (50 ml kg^−1^) immediately after wound closure and placed in cages with free access to food and water. The mice in the sham group underwent the same surgical procedures except CLP. After CLP, the mice were monitored for survival for 7 days. In a different set of experiments, blood and tissues were collected for cytokine measurements and histological analysis 4 and 8 h after CLP, respectively.

### Statistical analysis

The results are presented as means±s.d. Significant differences between two groups were determined using Student's *t*-test. The log-rank test was used to compare differences between survival curves. *P* values <0.05 were considered statistically significant.

### Data availability

The data that support the findings of this study are available from the corresponding author upon reasonable request.

## Additional information

**How to cite this article:** Lee, C.-Y. *et al*. The ubiquitin ligase ZNRF1 promotes caveolin-1 ubiquitination and degradation to modulate inflammation. *Nat. Commun.* **8**, 15502 doi: 10.1038/ncomms15502 (2017).

**Publisher's note:** Springer Nature remains neutral with regard to jurisdictional claims in published maps and institutional affiliations.

## Supplementary Material

Supplementary Information Supplementary figures and supplementary tables.
