# Supplementary material for: The ubiquitin ligase ZNRF1 promotes caveolin-1 ubiquitination and degradation to modulate inflammation
Source: Nat Commun. 2017 Jun 8;8:15502. doi: 10.1038/ncomms15502 (PMC5472178; doi:10.1038/ncomms15502)
Supplement: Supplementary Information — Supplementary figures and supplementary tables. [file ncomms15502-s1.pdf]

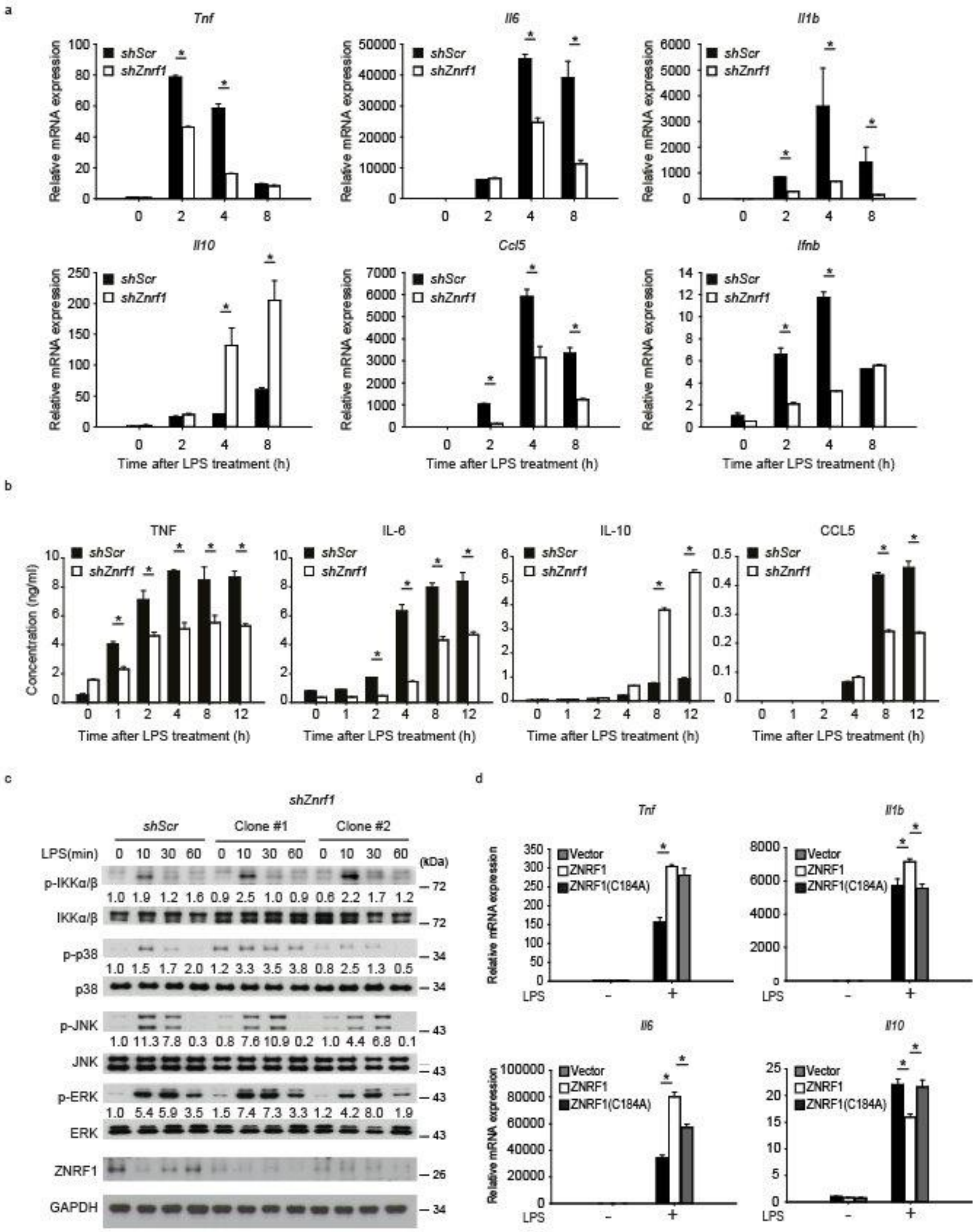

Supplementary Figure 1. Depletion of ZNRF1 in RAW264.7 cells significantly inhibits LPS-induced inflammatory responses.

(a-c) RAW264.7 macrophages infected with lentiviruses expressing control shRNA (*shScr*) or *shZnrf1* were treated with LPS (100 ng/ml) for the indicated times. (a) The mRNA expression of the indicated genes was determined by RT-qPCR, and (b) the production of cytokines in supernatants was analyzed by ELISA. (c) Immunoblot analysis of the phosphorylation of MAPKs

and IKKα/β as well as the indicated proteins in cell lysates. The intensities of the bands are shown as fold increases compared to those of untreated control cells after normalization to their unphosphorylated forms. (d) BMDMs from *Znrf1*$^{\Delta}$ mice were reconstituted with either Flag-tagged wild-type ZNRF1 or ZNRF1(C184A) mutant and stimulated with LPS (100 ng/ml) for 4 h (*Tnf* and *Il6)* or 8 h (*Il1b* and *Il10*). The expression of the indicated mRNAs was measured by RT-qPCR. *P<0.05 (Student's *t*-test). The data are representative of three independent experiments performed in triplicate (error bars, SD.).

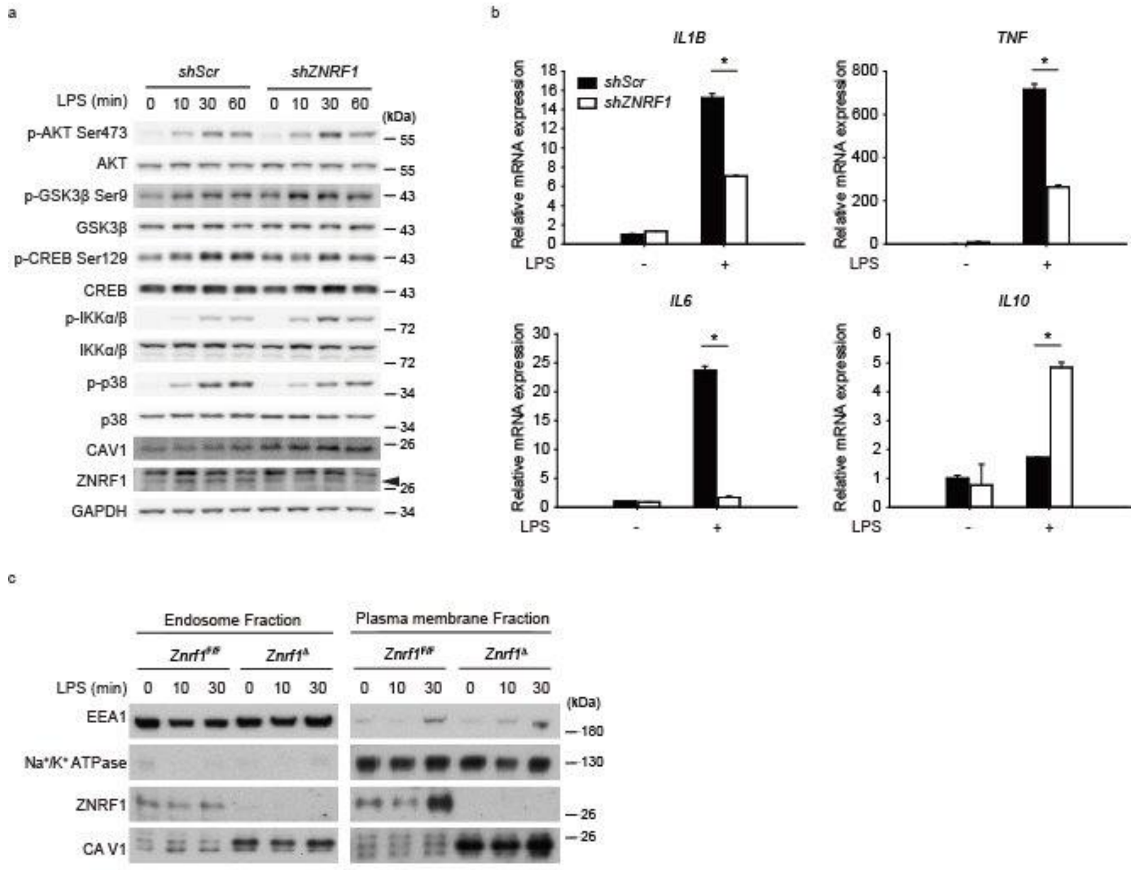

Supplementary Figure 2. Depletion of ZNRF1 in THP1 macrophages blunts LPS-induced inflammatory response.

(a and b) THP1 macrophages expressing control shRNA (*shScr*) or *shZNRF1* were stimulated with LPS (100 ng/ml) for the indicated times. (a) Cell lysates were analyzed by immunoblotting with indicated antibodies. The arrow indicates ZNRF1. (b) The expression of the indicated mRNAs was analyzed by RT-qPCR. *P<0.05 (Student's *t*-test). (c) BMDMs from *Znrf1*$^{\Delta}$ and *Znrf1*$^{F/F}$ mice were stimulated with LPS (100 ng/ml) for the indicated times. Cell lysates were collected and fractionated followed by immunoblot analysis with the indicated antibodies. EEA1 and Na^+/K^+ ATPase served as markers for the endosomes and the plasma membrane, respectively. The data are representative of three independent experiments performed in triplicate (error bars, SD.).

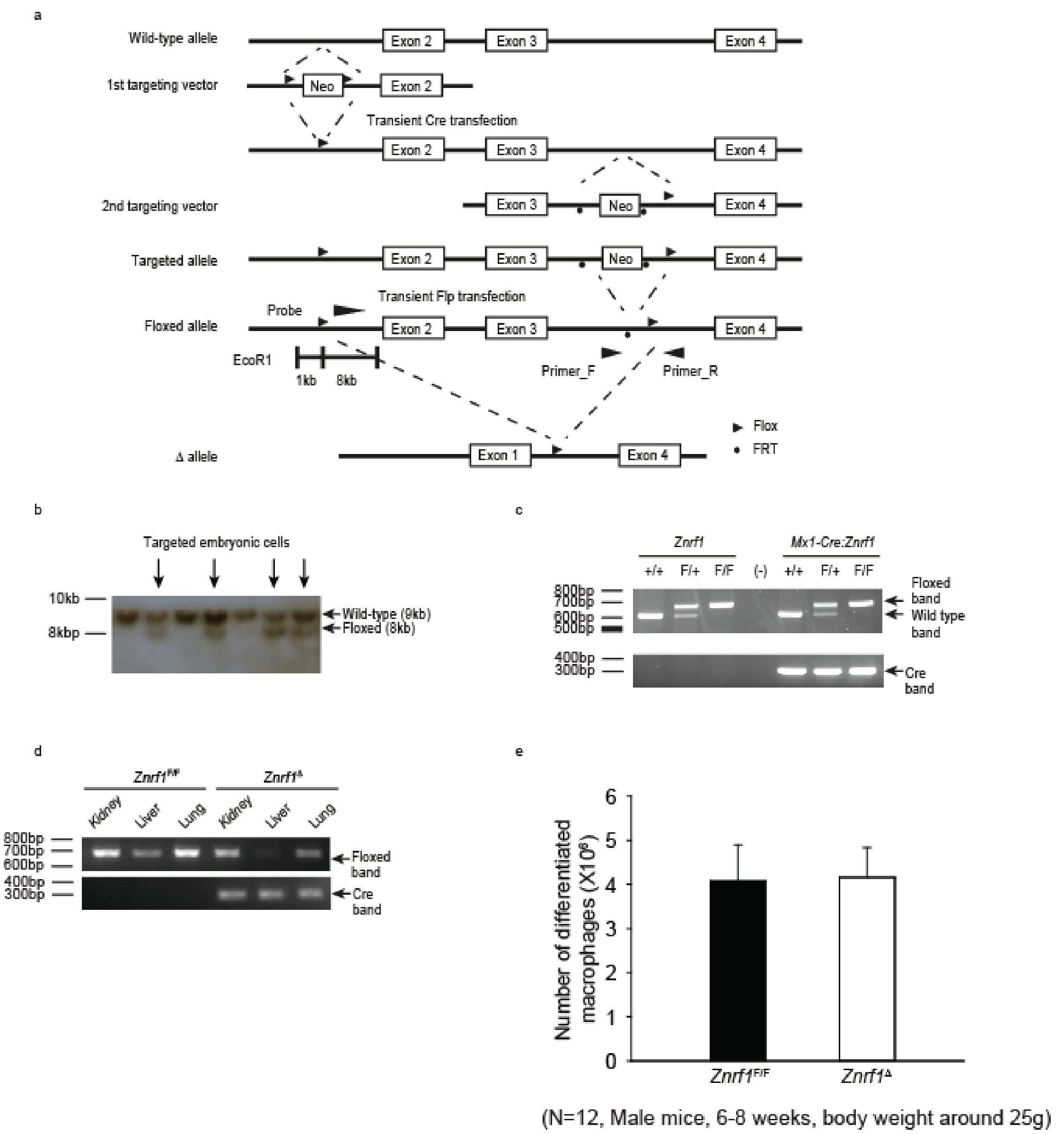

Supplementary Figure 3. Generation of mice lacking ZNRF1 in hematopoietic cells.

(a) Schematic diagram of the mouse wild-type *Znrf1* allele, the two targeting constructs, *Znrf1*^{Flox}-targeted alleles, and the deleted (Δ) *Znrf1* allele after *Cre*-mediated recombination. The initial targeting of the *Znrf1* allele created a new EcoRI restriction site in the first intron. The new EcoRI site resulted in a smaller EcoRI-digested fragment in Southern blot analysis. Arrowheads indicate the positions of the two primers used for PCR genotyping. (b) Southern blot genotyping of EcoRI-digested genomic DNA from targeted embryonic stem cells. (c and d) Genotyping of *Znrf1* alleles and *Cre* transgene by PCR using genomic DNA isolated from mouse (c) tails and (d) kidneys, livers, and lungs. (e) ZNRF1 deficiency has no obvious effect on the *in vitro*

differentiation of bone marrow cells into macrophages. The average cell numbers of bone marrow-derived macrophages from *Znrf1*$^{\Delta}$ and *Znrf1*$^{F/F}$ mice (n=12) are shown.

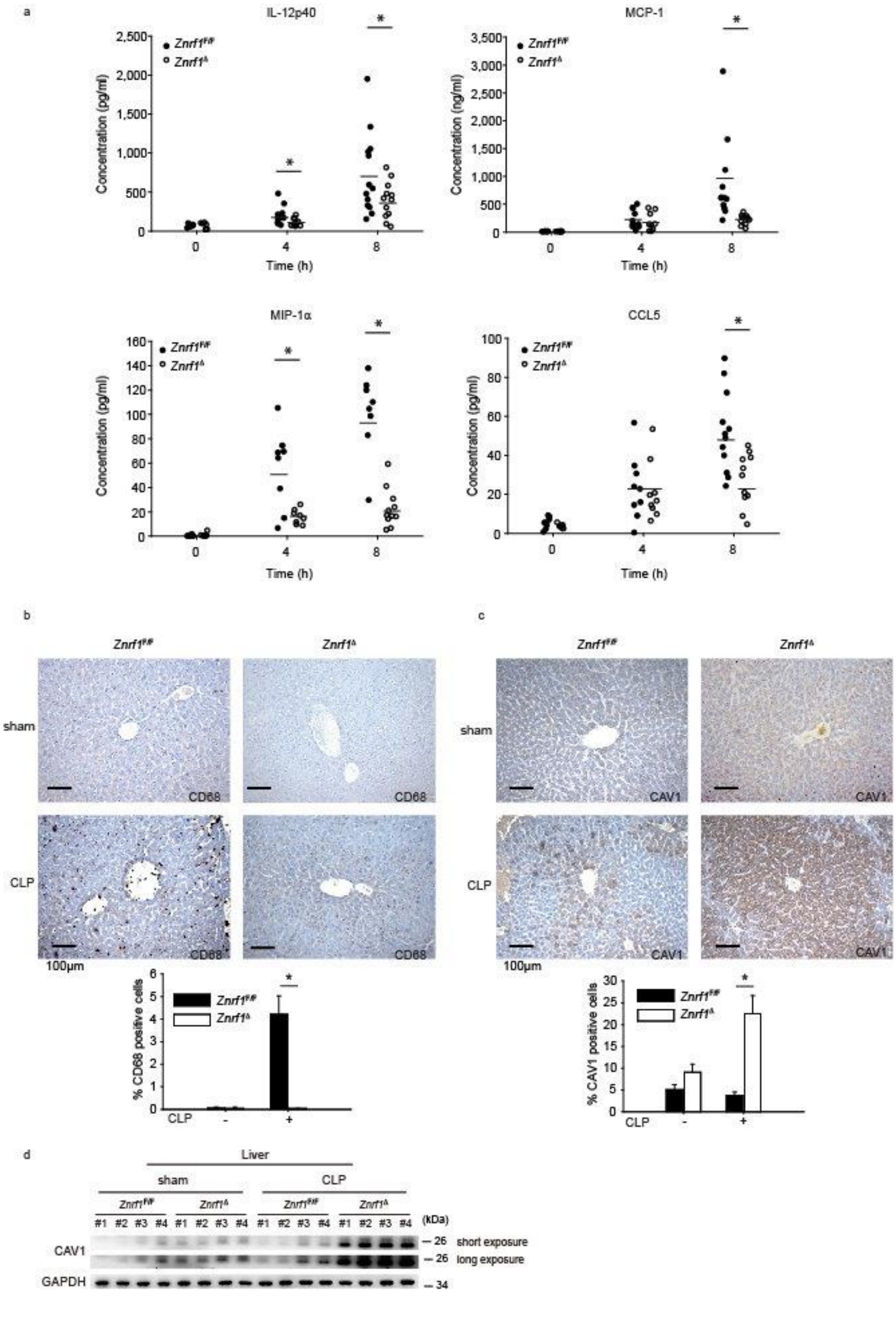
a
IL-12p40
MCP-1
MIP-1α
CCL5
Znrf1F/F
Znrf1Δ
Concentration (pg/ml)
Concentration (ng/ml)
Time (h)
b
c
sham
CLP
CD68
CAV1
100μm
% CD68 positive cells
% CAV1 positive cells
d
Liver
GAPDH
short exposure
long exposure
(kDa)

Supplementary Figure 4. Production of serum inflammatory cytokines and chemokines and immunohistochemical staining of liver tissues from CLP- challenged *Znrf1*$^{F/F}$ and *Znrf1*$^{\Delta}$ mice.

(a) Sera were collected from *Znrf1*$^{\Delta}$ and *Znrf1*$^{F/F}$ mice 8 h after CLP, and the levels of IL12p40, MCP-1, MIP-1α and CCL5 were measured by ELISA. (b and c) Liver tissues from *Znrf1*$^{\Delta}$ and *Znrf1*$^{F/F}$ mice (lower panel) 8 h after CLP and sham control (upper panel) were subjected to immunohistochemical staining for (b) CD68 and (c) CAV1 (objective magnification ×20). Scale bar is 100 μm. The total numbers of CD68^{+} and CAV1^{+} cells per field were quantified and are shown in the lower panel. (Error bars, SD. from 10 fields of view on 10 sections from liver per mouse, n=4 mice per group). *P<0.05 (Student's *t*-test). (d) CAV1 protein level in liver from *Znrf1*$^{\Delta}$ and *Znrf1*$^{F/F}$ mice 8 h after CLP or sham operation were examined by immunoblotting. (n=4 mice per group)

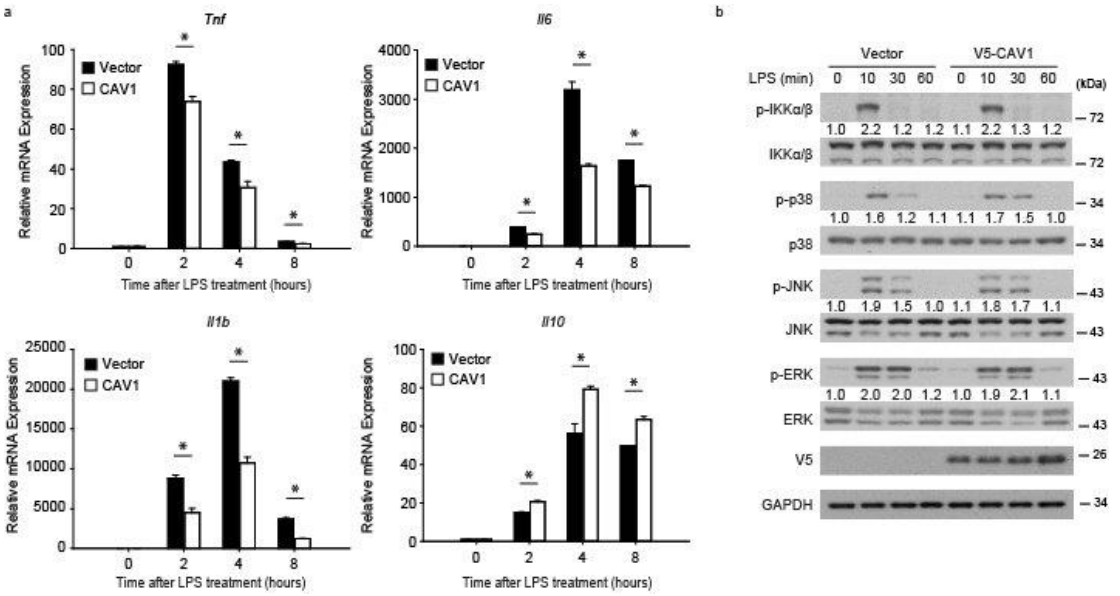

Supplementary Figure 5. Over-expression of CAV1 attenuates LPS-induced inflammatory responses in RAW264.7 macrophages.

(a and b) Control and CAV1-expressing RAW264.7 cells were stimulated with LPS (100 ng/ml) for the indicated times. (a) The mRNA expression levels of IL-1β, TNF, IL-6, and IL-10 were evaluated by RT-qPCR. (b) The activation of IKKα/β and MAPKs was detected by immunoblotting. The intensities of the bands are shown as fold increases compared to those of untreated control cells after normalization to their unphosphorylated forms. *P<0.05 (Student's *t*-test). The data are representative of three independent experiments performed in triplicate (error bars, SD.).

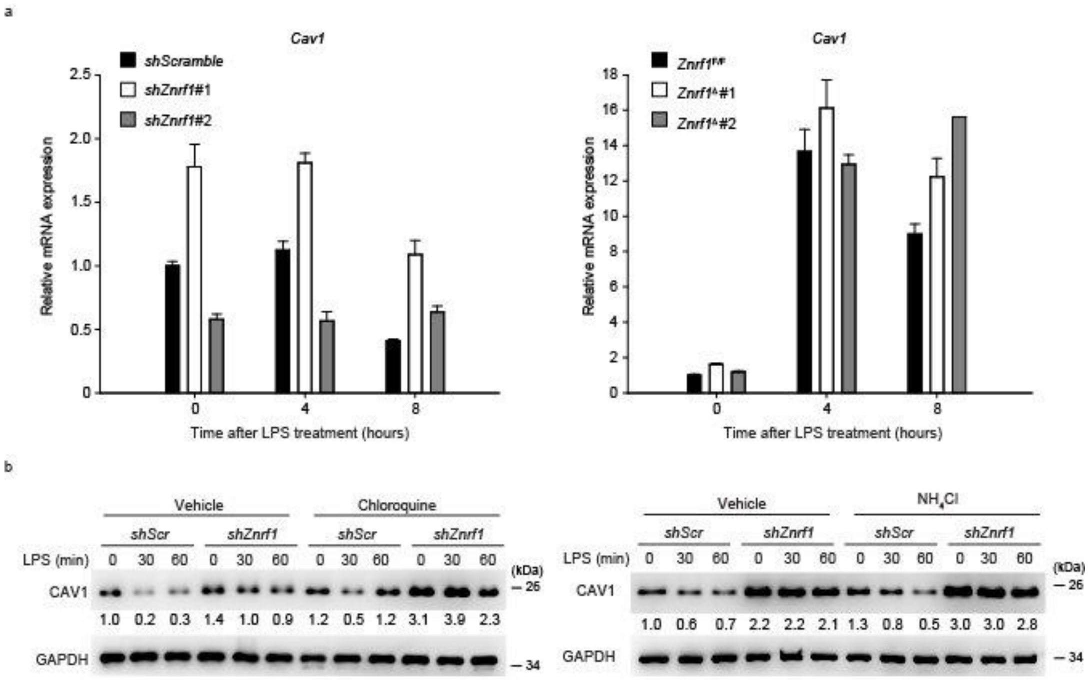

Supplementary Figure 6. The endolysosomal pathway plays a minimal role in ZNRF1-controlled CAV1 protein expression.

(a) The mRNA expression levels of CAV1 in control and *shZnrf1*-expressing RAW264.7 cells (left) and in BMDMs from *Znrf1*$^{F/F}$ and *Znrf1*$^{\Delta}$ mice (right) treated with LPS (100 ng/ml) for the indicated times was determined by RT-qPCR. *P<0.05 (Student's *t*-test). (b) Control and *shZnrf1*-expressing RAW264.7 cells pretreated with chloroquine (100 μM) or NH_4Cl (10 mM) were incubated with LPS (100 ng/ml) for the indicated times. CAV1 protein expression was analyzed by immunoblotting. The intensities of the bands are shown as fold increases compared to those of untreated control cells after normalization to GAPDH expression. The data are representative of two or three independent experiments performed in triplicate (error bars, SD.).

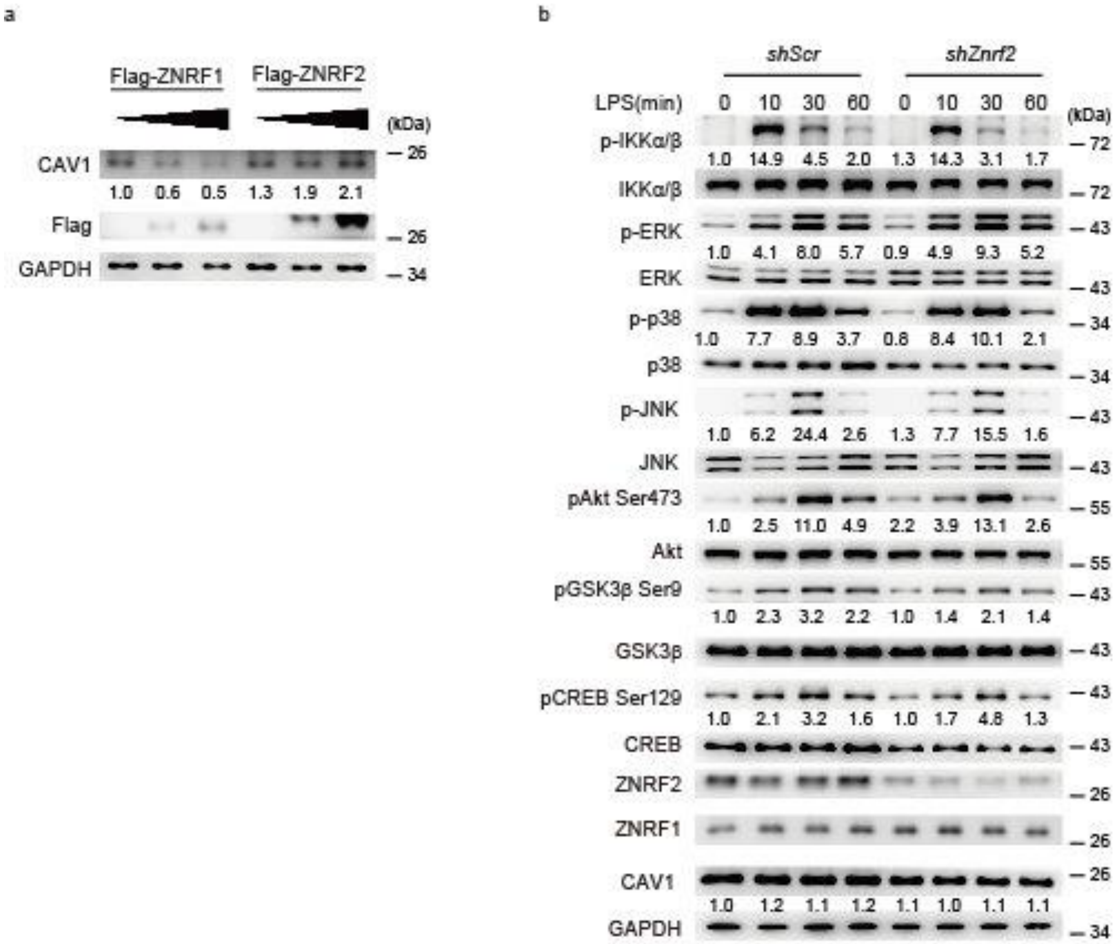

Supplementary Figure 7. ZNRF2 does not regulate CAV1 protein level and TLR4-driven inflammatory response

(a) HEK293T cells were transfected with increasing amounts of Flag-tagged wild-type ZNRF1 or ZNRF2; 24 h after transfection, cell lysates were analyzed by immunoblotting with the indicated antibodies. (b) RAW264.7 macrophages infected with lentiviruses expressing *shScr* or *shZnrf2* were stimulated with LPS (100 ng/ml) for the indicated times. Cell lystaes were analyzed by immunoblotting with the indicated antibodies. The intensities of the bands are shown as fold increases compared to those of untreated control cells after normalization to their unphosphorylated forms or GAPDH expression. The data are representative of three independent experiments.

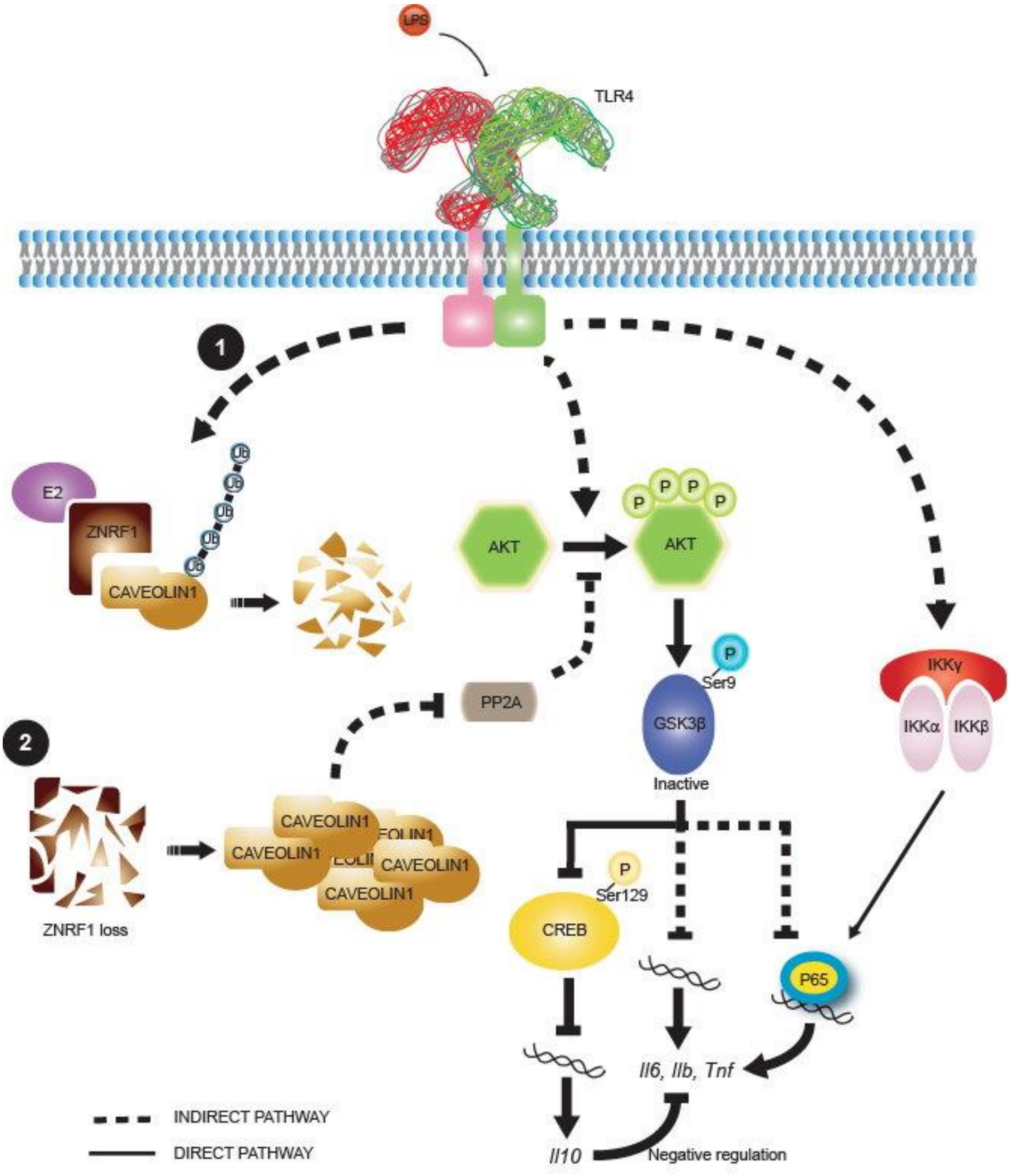

Supplementary Figure 8. A proposed model summarizing the control of CAV1 protein level and TLR-triggered immune responses by ZNRF1.

TLR4 activation induces an interaction between ZNRF1 and CAV1, which promotes CAV1 ubiquitination and degradation through the proteasome pathway, thereby shifting the balance of the immune response toward increased pro-inflammatory cytokine production. When ZNRF1 is depleted, the elevated expression of CAV1 increases Akt phosphorylation and activation, resulting in the inactivation of GSK3β by phosphorylation at serine 9. This leads to reduced phosphorylation of CREB serine 129, which results in a substantial increase in IL-10 expression

and blocks the production of pro-inflammatory cytokines by suppressing the nuclear translocation of transcription factor NF-κB subunit p65.

Figure 1c

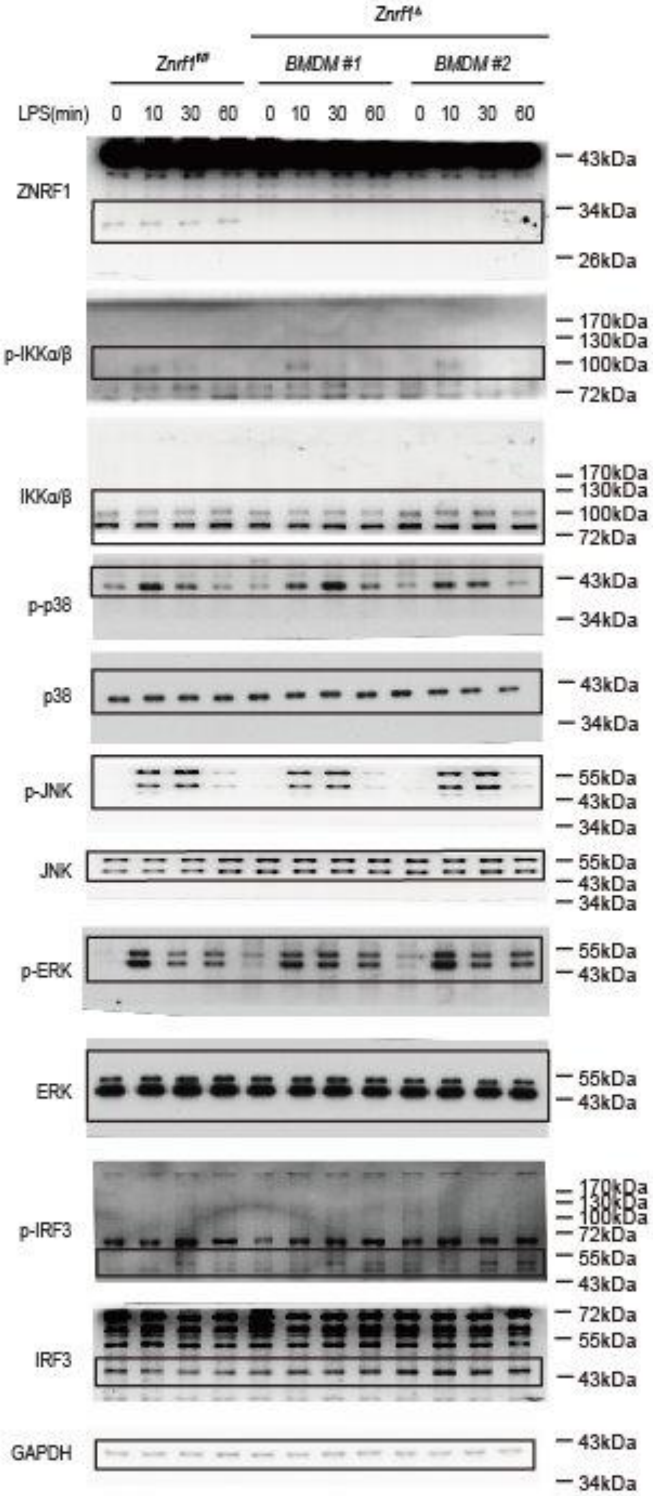

Figure 1d

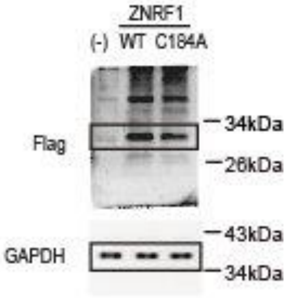

Figure 3a

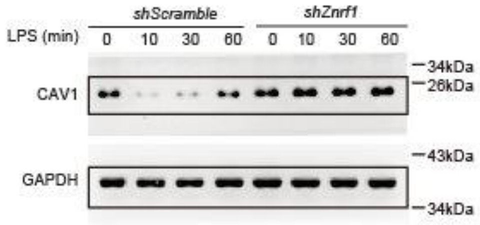

Figure 3b

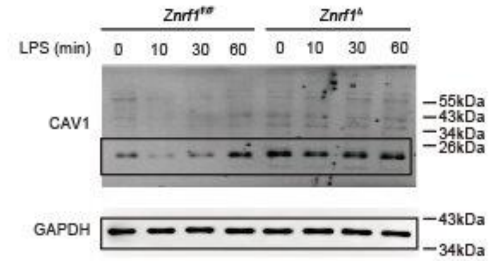

Figure 3c

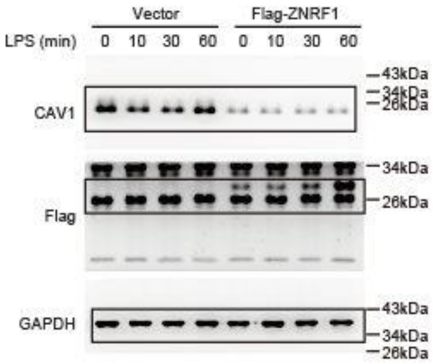

Figure 3d

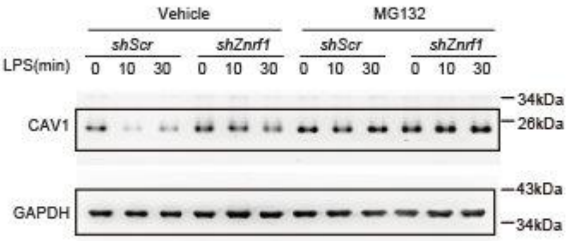

Figure 3e

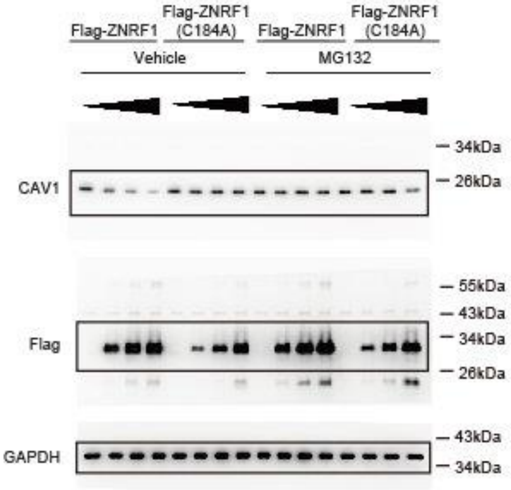

Figure 4a

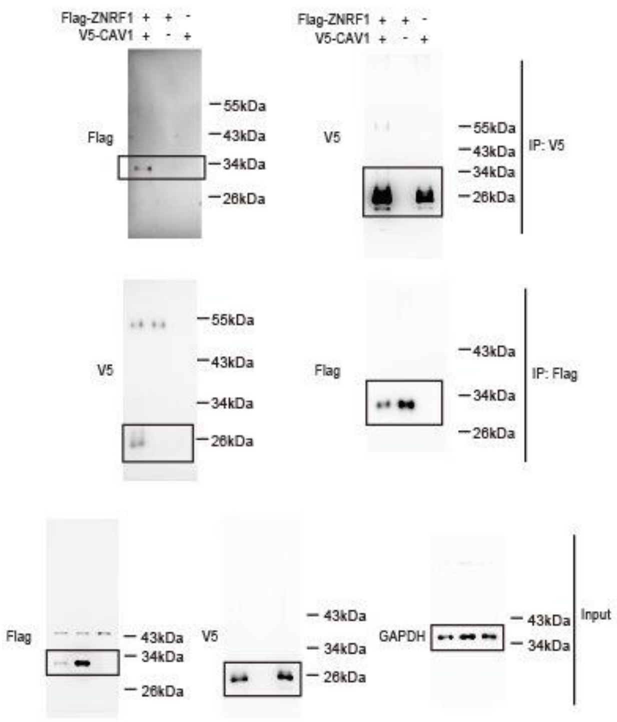

Figure 4b

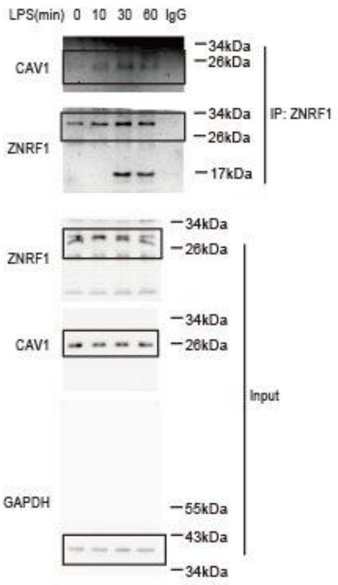

Figure 4d

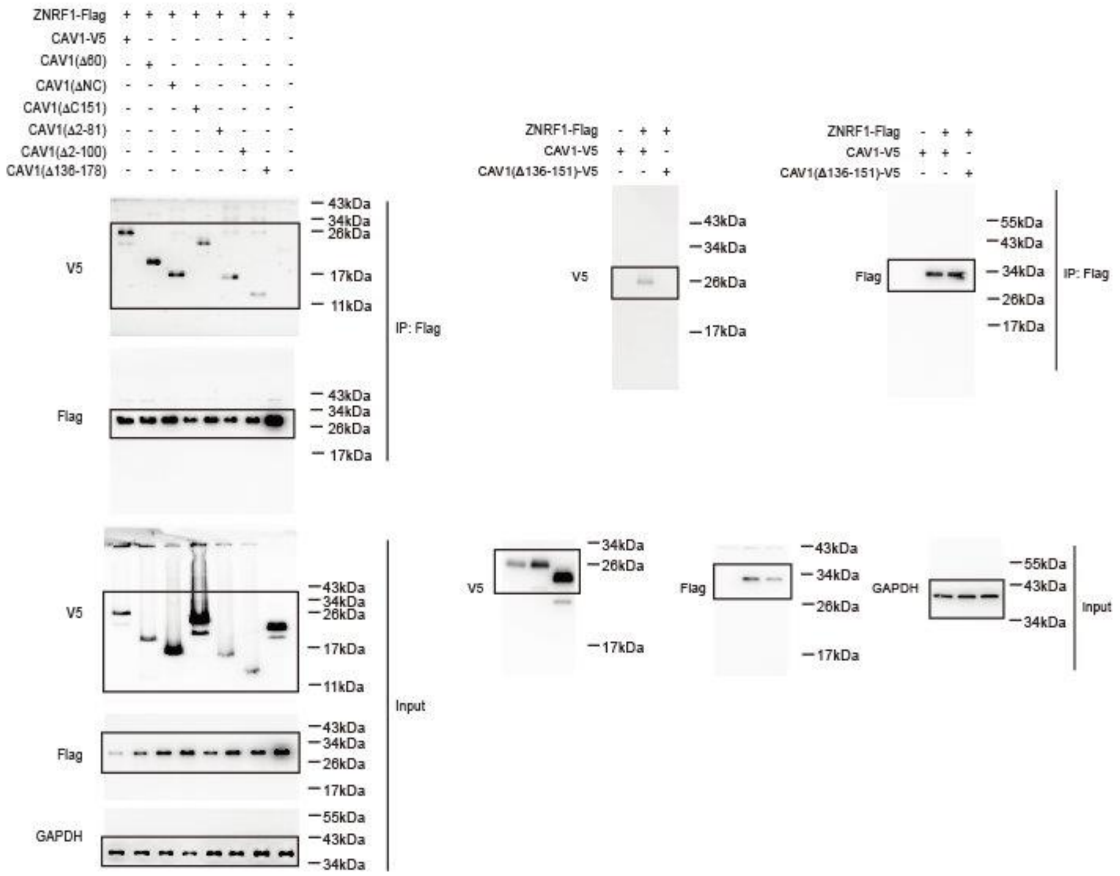

Figure 4f

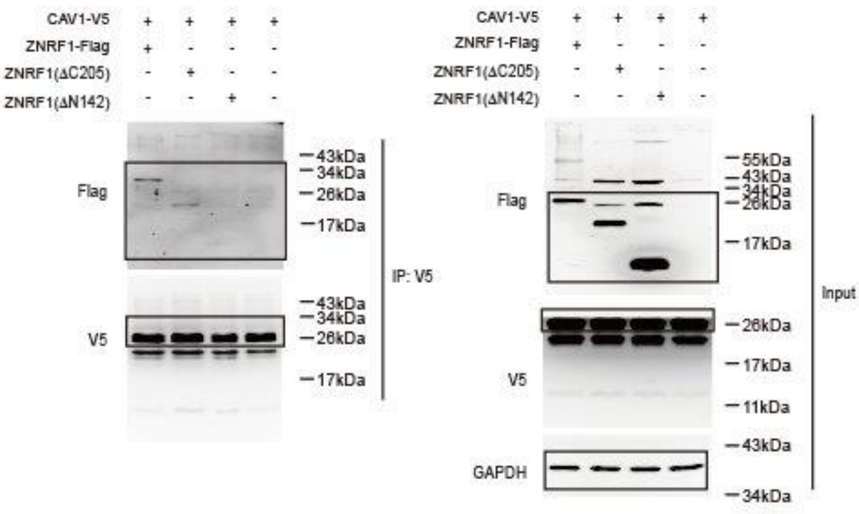

Figure 4g

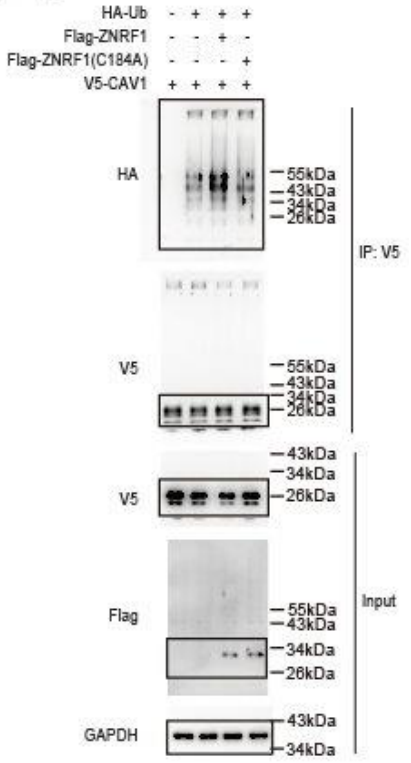

Figure 4h

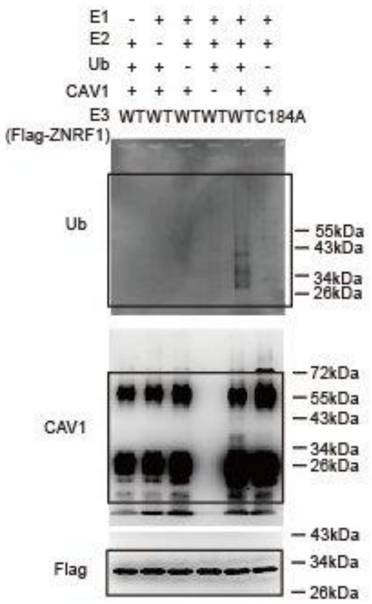

Figure 4i

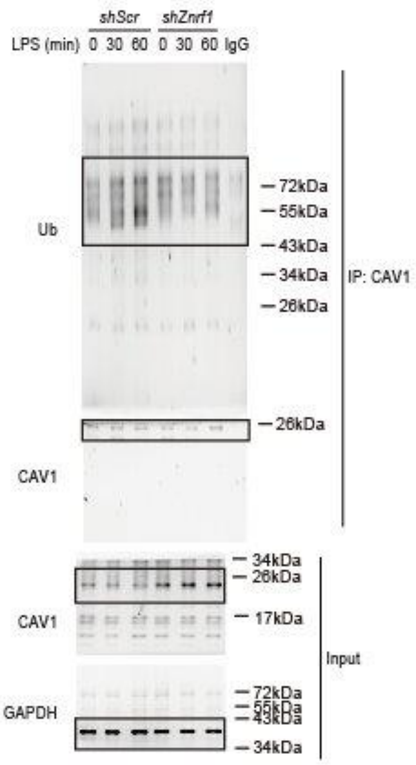

Figure 4j

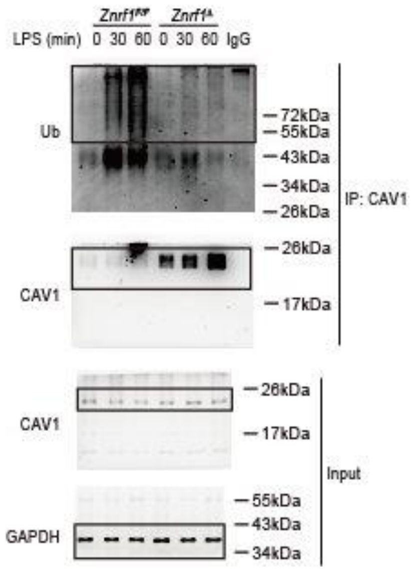

Figure 5b

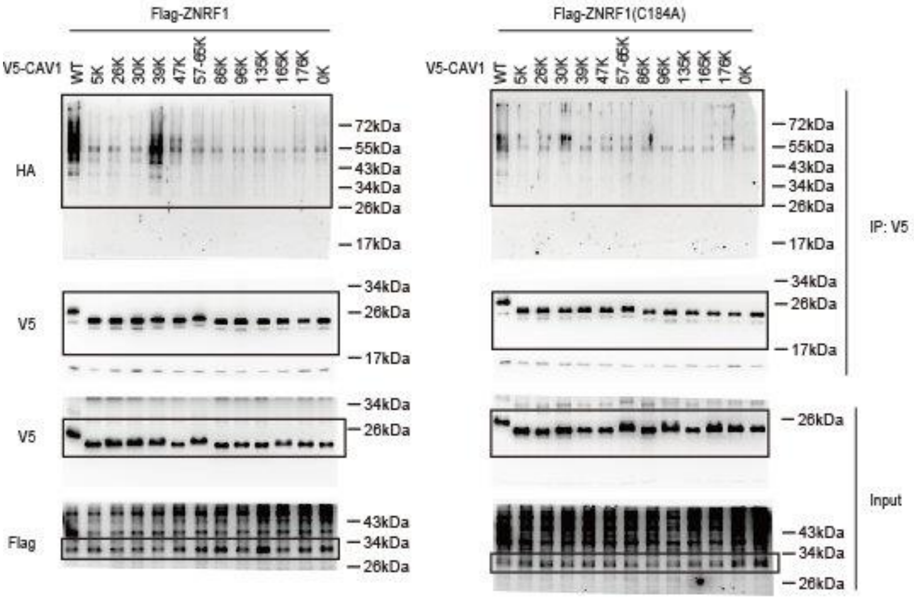

Figure 5c

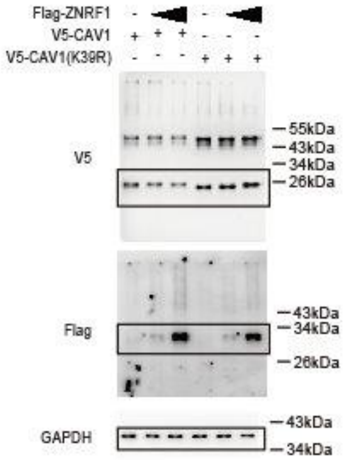

Figure 5d

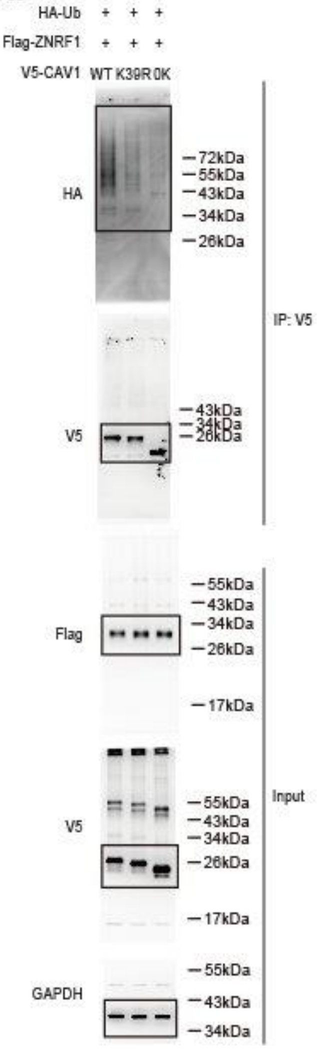

Figure 5e

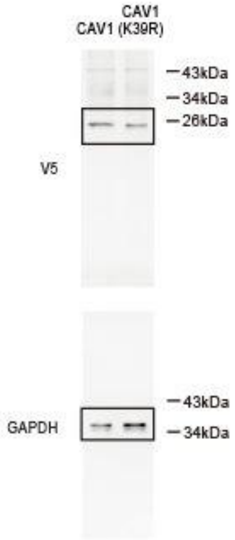

Figure 6a

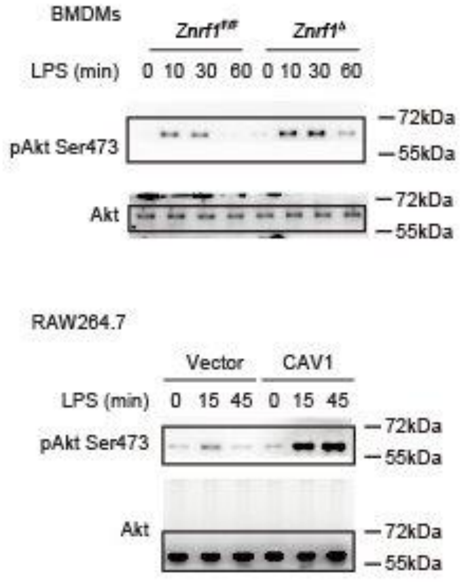

Figure 6c

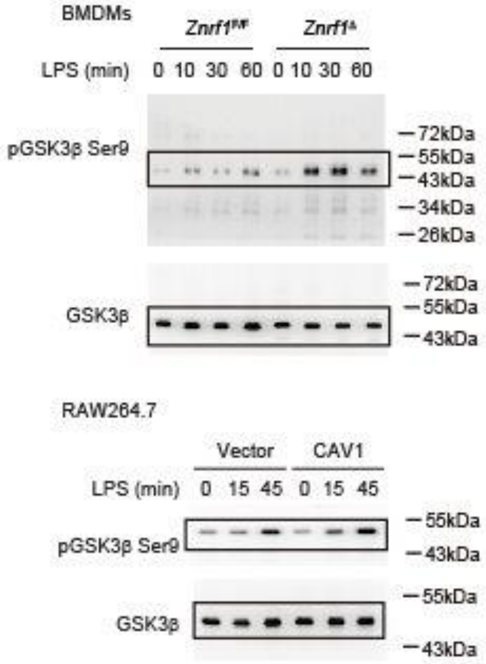

Figure 6d

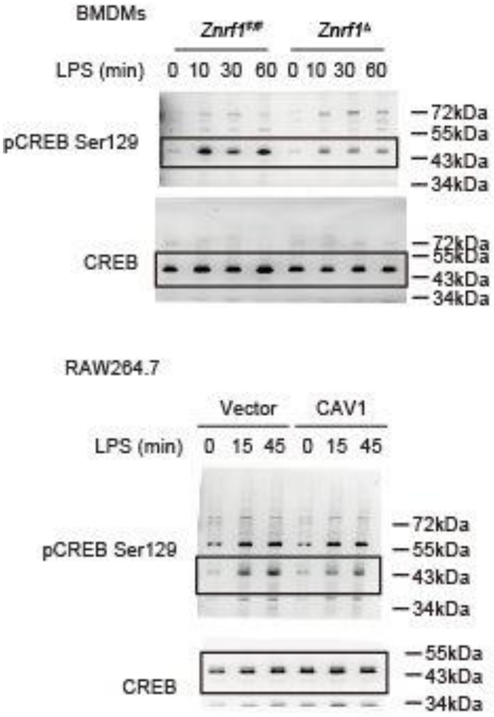

Figure 6e

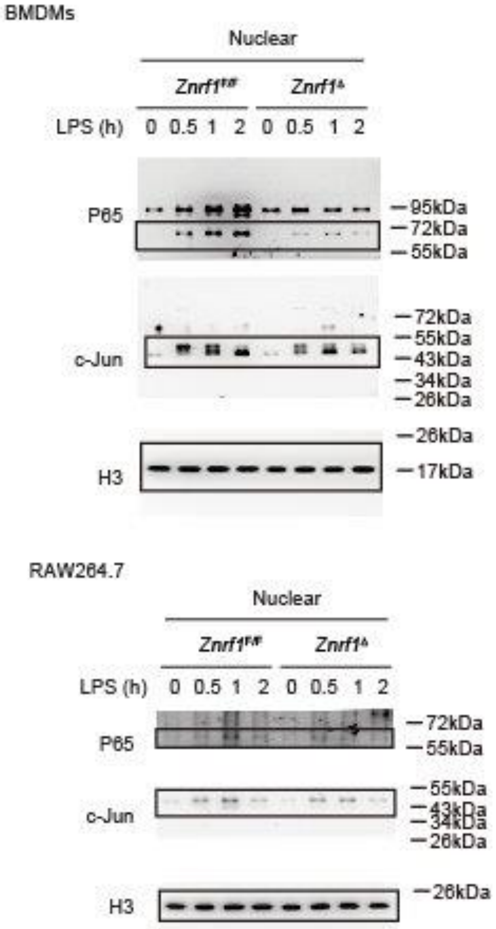

Figure 7c

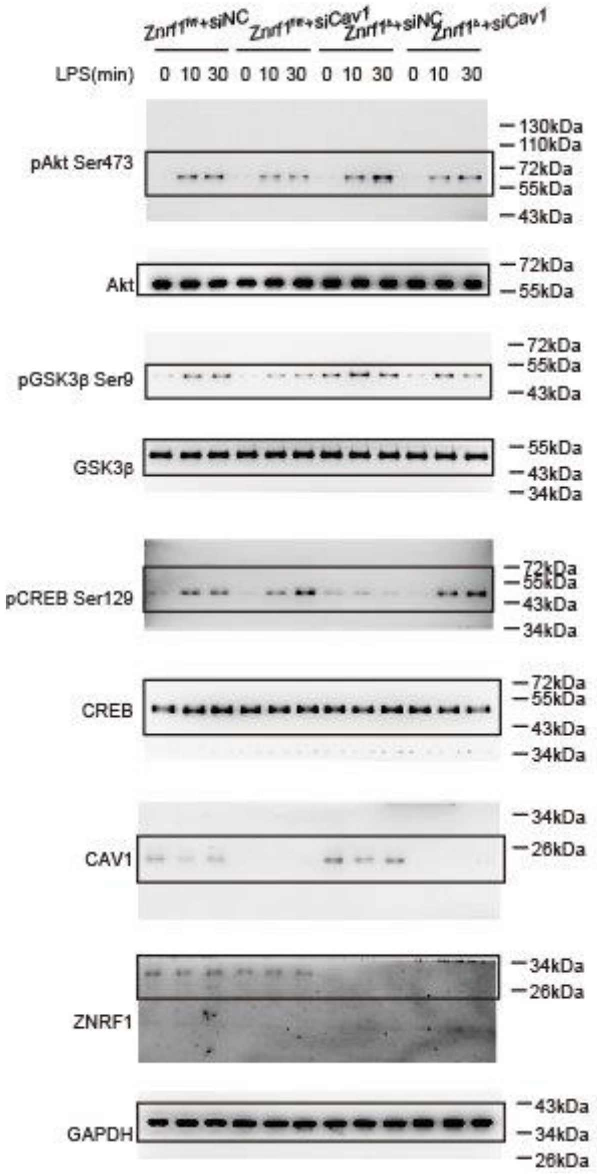

Figure 7d

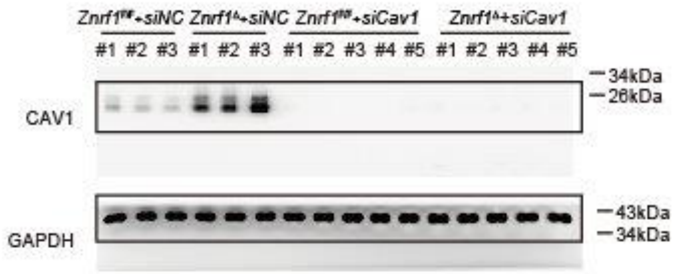

Supplementary Figure 1c

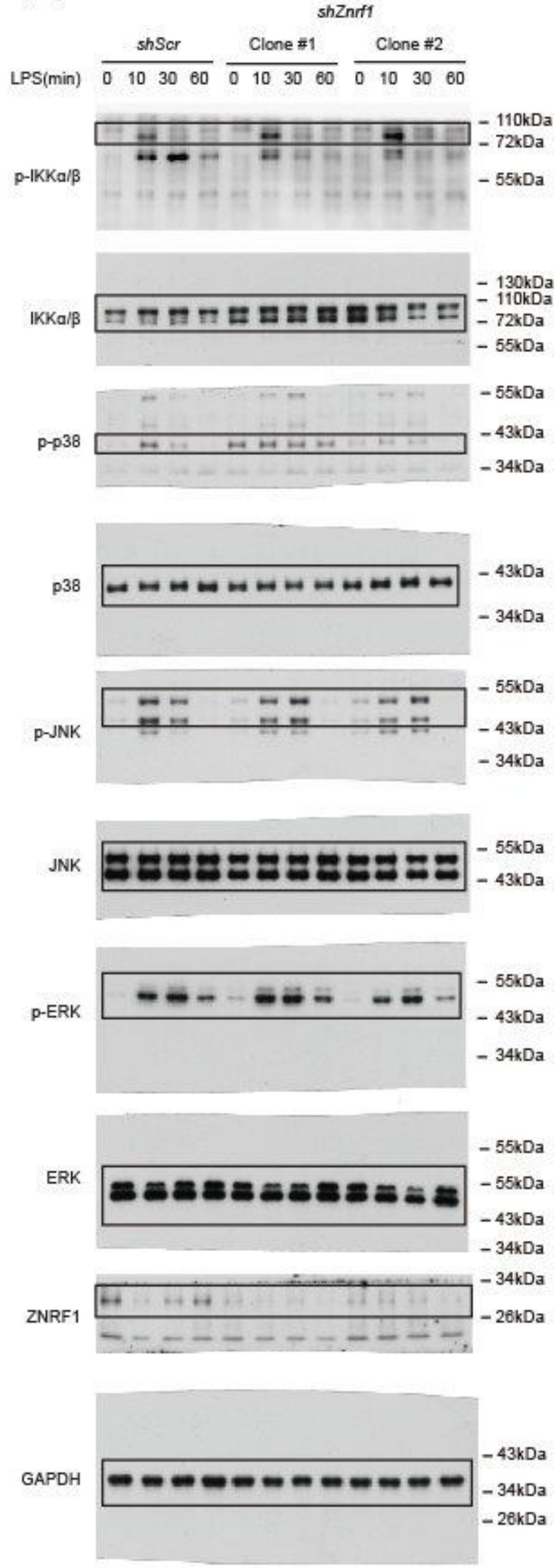

Supplementary Figure 2a

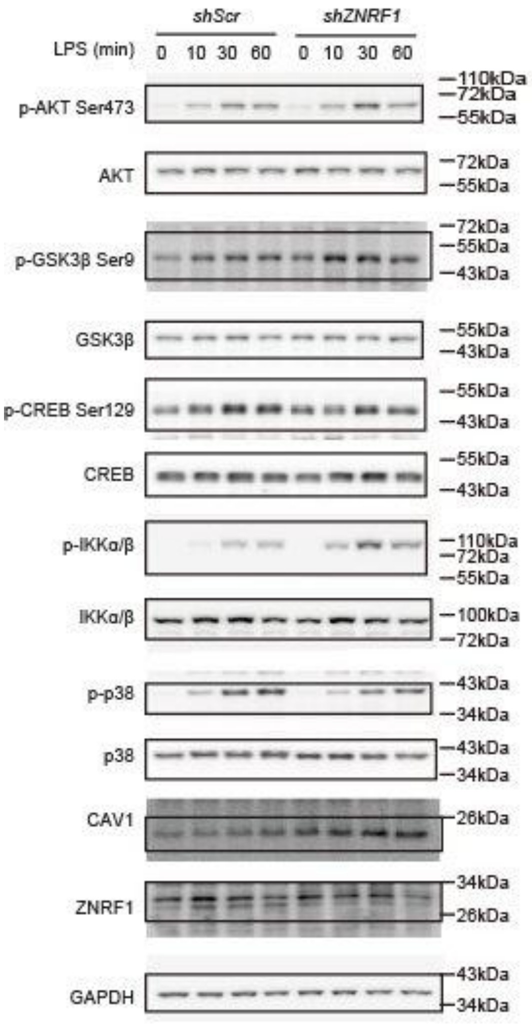

Supplementary Figure 2c

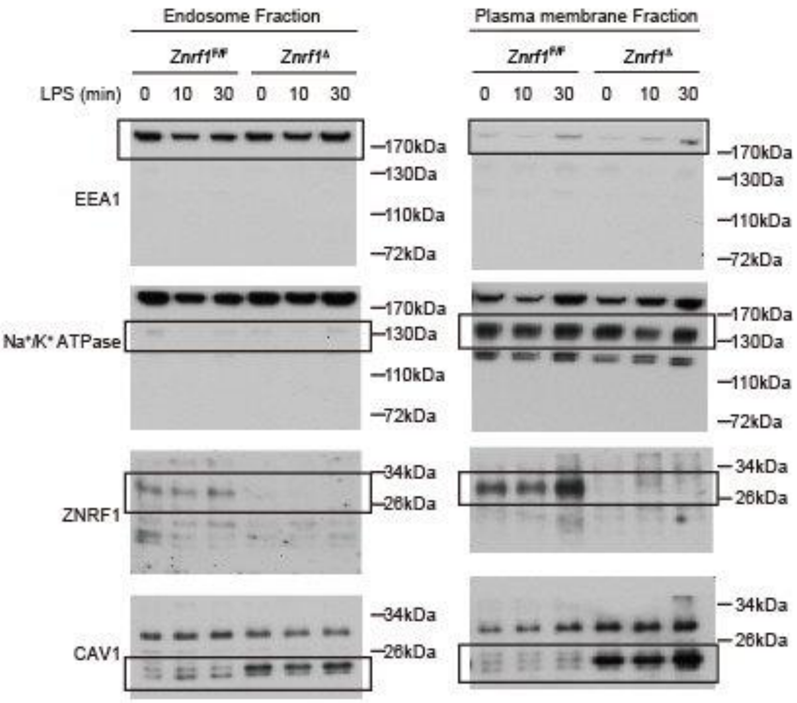

Supplementary Figure 3b

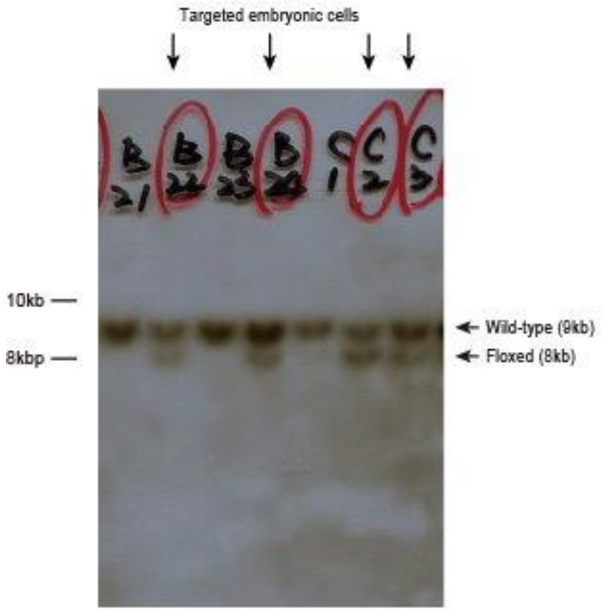

Supplementary Figure 3c

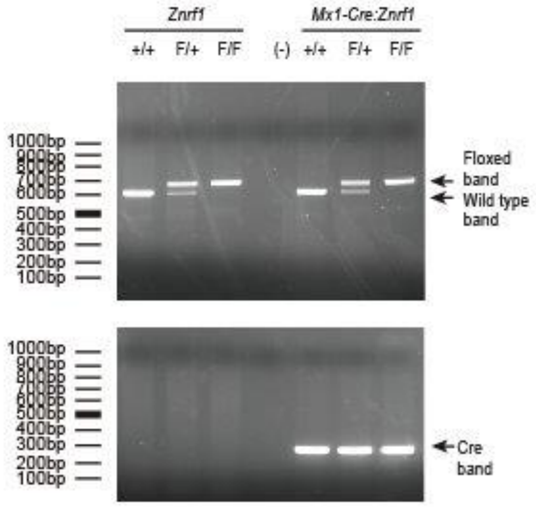

Supplementary Figure 3d

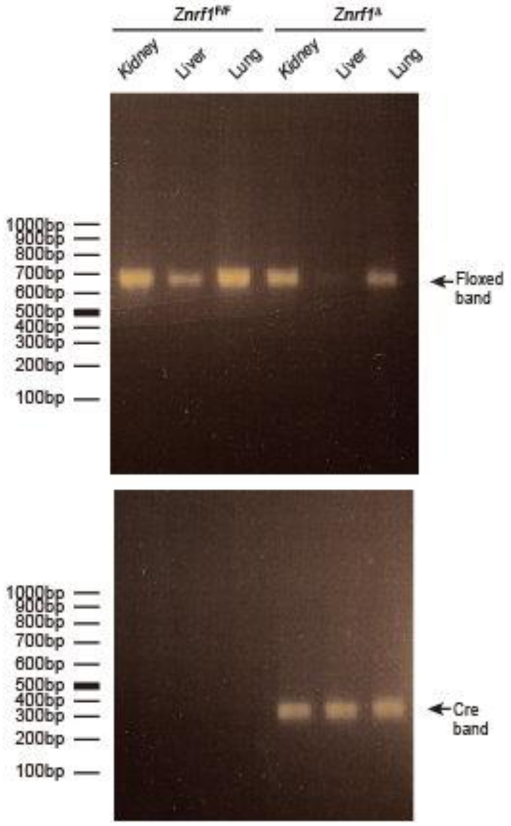

Supplementary Figure 4d

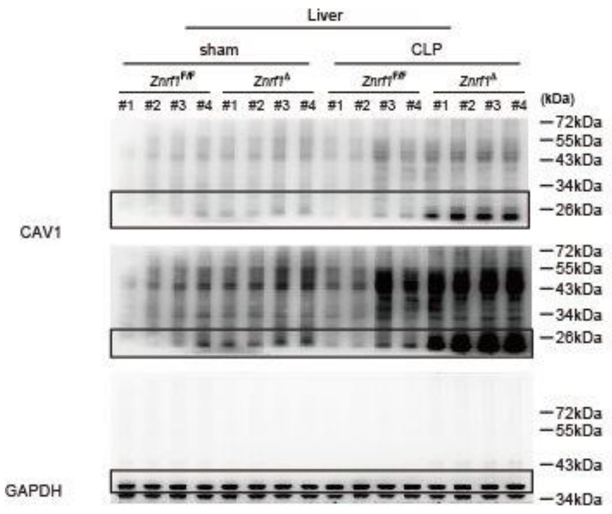

Supplementary figure 5b

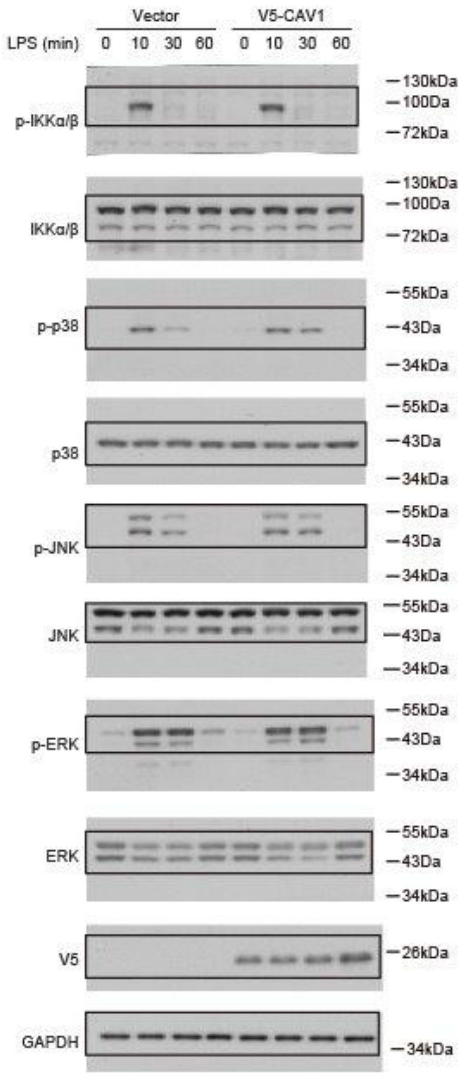

Supplementary Figure 6b

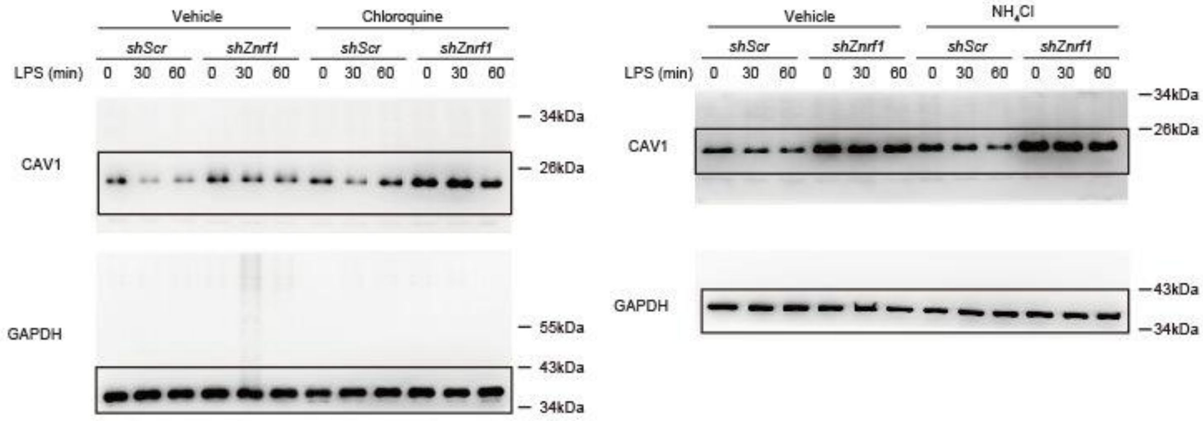

Supplementary Figure 7a

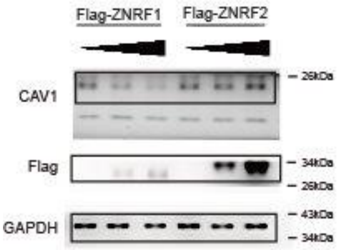

Supplementary Figure 7b

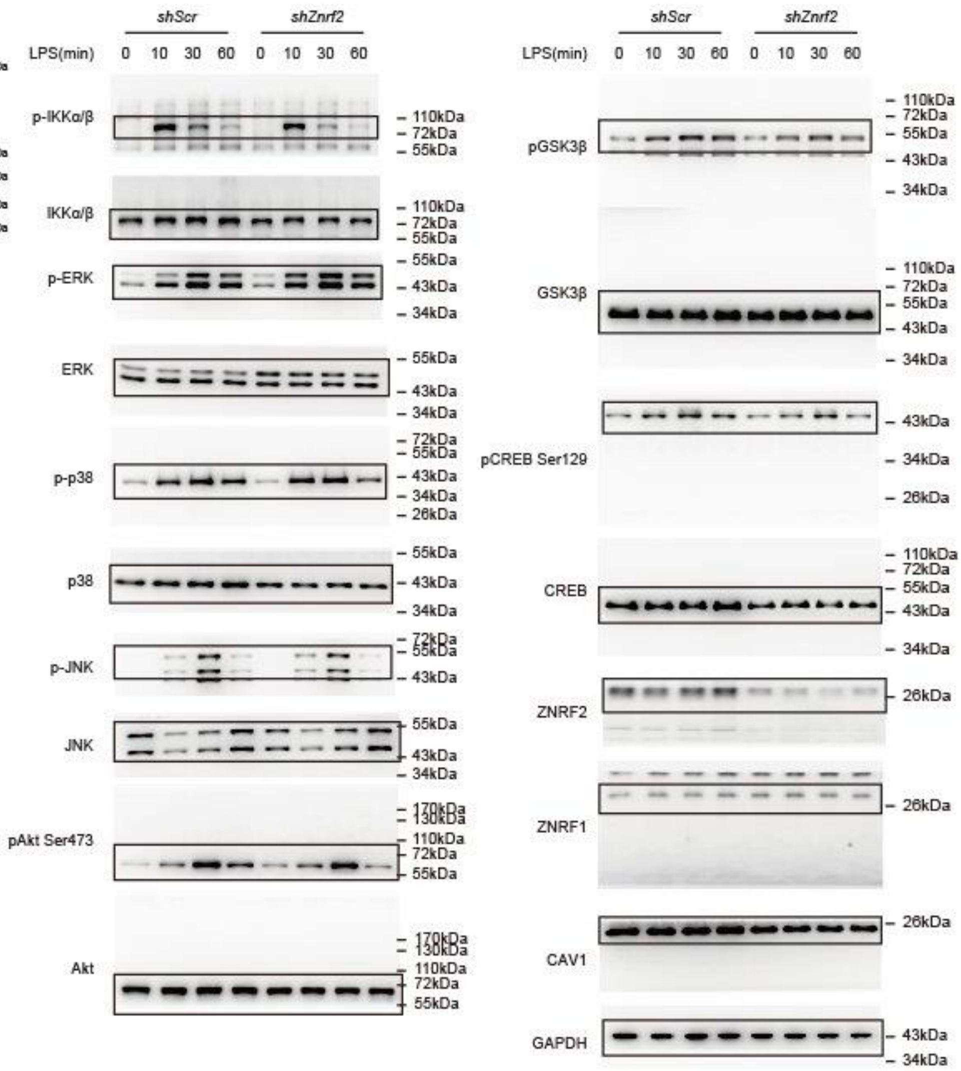

Supplementary Figure 9. Uncropped scans of the blot images shown in Figures.

Supplementary Table 1

Primer pairs for RT-qPCR

Mouse *Cyclophilin* Forward:	ATGTGCCAGGGTGGTGACTTT
Mouse *Cyclophilin* Reverse:	TTGCCATCCAGCCATTCAGTC
Mouse *Tnf* Forward:	GTCTACTGAACTTCGGGGTGATC
Mouse *Tnf* Reverse:	TCCACTTGGTGGTTTGCTACG
Mouse *Il6* Forward:	ACAAGAAAGACAAAGCCAGAGTC
Mouse *Il6* Reverse:	ATTGGAAATTGGGGTAGGAAG
Mouse *Il10* Forward:	TGGGTTGCCAAGCCTTATCGG
Mouse *Il10* Reverse:	ACCTGCTCCACTGCCTTGCTC
Mouse *Il1b* Forward:	GAACTCAACTGTGAAATGCCACC
Mouse *Il1b* Reverse:	CCACAGCCACAATGAGTGATACT
Mouse *Ccl5* Forward:	GACACCACTCCCTGCTGCTTTG
Mouse *Ccl5* Reverse:	GATGTATTCTTGAACCCACTTCTT
Mouse *Ifnb* Forward:	GCTGCGTTCCTGCTGTGCTTCT
Mouse *Ifnb* Reverse:	CGCCCTGTAGGTGAGGTTGATC
Mouse *Cav1* Forward:	CTTCGGCATCCCAATGGCACTC
Mouse *Cav1* Reverse:	AGGTATGGACGTAGATGGAGTA
Human *CYCLOPHILIN* Forward:	ATACGGGTCCTGGCATCTTGTC
Human *CYCLOPHILIN* Reverse:	GGTGATCTTCTTGCTGGTCTTG

Human *TNF* Forward:	CCTGCTGCACTTTGGAGTGATC
Human *TNF* Reverse:	ACTCGGGGTTCGAGAAGATGAT
Human *IL6* Forward:	TGCTTCCAATCTGGATTCAATG
Human *IL6* Reverse:	GGTTGGGTCAGGGGTGGTTATT
Human *IL10* Forward:	CTGAGAACCAAGACCCAGACAT
Human *IL10* Reverse:	AGGCATTCTTCACCTGCTCCAC
Human *IL1B* Forward:	CGAATCTCCGACCACCACTAC
Human *IL1B* Reverse:	GCACATAAGCCTCGTTATCCC

Supplementary Table 2

Complete blood counts of *Znrf1*$^{\Delta}$ and *Znrf1*$^{F/F}$ mice

Parameters	*Znrf1*$^{F/F}$	*Znrf1*$^{\Delta}$	P-value
RBC ($10^6/mm^3$)	8.75±0.36	8.82±0.31	0.78
Hb (g/dl)	13.9±0.51	13.8±0.51	0.78
WBC ($10^3/mm^3$)	2.37±0.86	2.99±0.86	0.22
Lymphocytes (%)	76.61±3.8	77.46±6.83	0.76
Granulocytes (%)	11.68±0.85	12.98±1.89	0.17

The data represent the average values (mean±SD.) for six *Znrf1*$^{\Delta}$ and *Znrf1*$^{F/F}$ mice examined 2 weeks after two poly(I:C) inductions (10 μg/g of body weight on day 0 and day 2).
